# Association of the Frequency and Quantity of Alcohol Consumption With Gastrointestinal Cancer

**DOI:** 10.1001/jamanetworkopen.2021.20382

**Published:** 2021-08-18

**Authors:** Jung Eun Yoo, Dong Wook Shin, Kyungdo Han, Dahye Kim, Su-Min Jeong, Hye Yeon Koo, Su Jong Yu, Jinsung Park, Kui Son Choi

**Affiliations:** 1 Department of Family Medicine, Healthcare System Gangnam Center, Seoul National University Hospital, Seoul, Republic of Korea; 2 Supportive Care Center, Department of Family Medicine, Samsung Medical Center, Seoul, Republic of Korea; 3 Department of Clinical Research Design & Evaluation, Samsung Advanced Institute for Health Science & Technology, Sungkyunkwan University, Seoul, Republic of Korea; 4 Department of Statistics and Actuarial Science, Soongsil University, Seoul, Republic of Korea; 5 Department of Medical Statistics, The Catholic University of Korea, Seoul, Republic of Korea; 6 Department of Family Medicine, Seoul Metropolitan Government–Seoul National University Boramae Medical Center, Seoul, Republic of Korea; 7 Department of Family Medicine, Seoul National University Health Service Center, Seoul, Republic of Korea; 8 Department of Nutrition, Harvard T.H. Chan School of Public Health, Boston, Massachusetts; 9 Health Promotion Center, Cha Bundang Medical Center, Seongnam, Republic of Korea; 10 Department of Internal Medicine and Liver Research Institute, Seoul National University College of Medicine, Seoul National University Hospital, Seoul, Republic of Korea; 11 Biomedical Research Institute, Center for Medical Innovation, Seoul National University Hospital, Seoul, Republic of Korea; 12 Department of Urology, Uijeongbu Eulji Medical Center, Eulji University, Uijeongbu-si, Republic of Korea; 13 Department of Cancer Control and Population Health, Graduate School of Cancer Science and Policy, National Cancer Center, Goyang, Republic of Korea

## Abstract

**Importance:**

Although total alcohol consumption is a known risk factor for gastrointestinal (GI) cancers, few studies have attempted to assess the pattern of alcohol drinking in association with GI cancers.

**Objective:**

To evaluate the relative association of the frequency of drinking vs the amount of alcohol consumed per occasion with the development of GI cancers.

**Design, Setting, and Participants:**

A population-based retrospective cohort study used data from the Korean National Health Insurance System database on 11 737 467 participants without cancer who underwent a national health screening program from January 1, 2009, to December 31, 2010. Participants were followed up from the year after their health screening date until they received a diagnosis of GI cancer, death, or December 31, 2017. The median follow-up duration was 6.4 years (interquartile range, 6.4-7.4 years). Statistical analysis was performed from January 1, 2019, to March 31, 2020.

**Exposures:**

Weekly alcohol consumption (nondrinker [0 g/week], mild drinker [0-104 g/week], moderate drinker [105-209 g/week], and heavy drinker [≥210 g/week]), drinking frequency, and amount per occasion.

**Main Outcomes and Measures:**

Incident GI cancers at 6 specific sites (esophagus, stomach, colorectal, liver, biliary, and pancreas).

**Results:**

Among 11 737 467 participants (6 124 776 women [52.2%]; mean [SD] age, 54.6 [10.4] years), 319 202 (2.7%) developed GI cancer. Compared with nondrinkers, the risk of GI cancer was higher for mild drinkers (adjusted hazard ratio [aHR], 1.04; 95% CI, 1.03-1.05), moderate drinkers (aHR, 1.14; 95% CI, 1.12-1.15), and heavy drinkers (aHR, 1.28; 95% CI, 1.26-1.29). The risk of GI cancer increased linearly with the frequency of drinking in a dose-dependent manner (aHR, 1.39; 95% CI, 1.36-1.41 for individuals who drink every day). In contrast, the risk of GI cancer appeared to increase with consumption up to 5 to 7 units per occasion (aHR, 1.15; 95% CI, 1.14-1.16), and then the HRs were no higher for those with a higher intake per session than 5 to 7 units (8-14 units per occasion: aHR, 1.11; 95% CI, 1.09-1.12; >14 units per occasion: aHR, 1.11; 95% CI, 1.08-1.14). Given similar weekly alcohol consumption levels, the risk of GI cancer increased with a higher frequency of drinking and decreased with a higher amount per occasion. Risk patterns for 6 specific cancers were generally similar to that of all GI cancers.

**Conclusions and Relevance:**

In this cohort study, frequent drinking was a more important risk factor for incident GI cancers than the amount of alcohol consumed per occasion. Individuals should be cautioned about regular consumption of small amounts of alcohol in addition to the total amount of alcohol consumption or amount per occasion.

## Key Points

**Question:**

Which is the more important factor in the development of gastrointestinal (GI) cancer: frequency of drinking alcohol or quantity of alcohol consumed per occasion?

**Findings:**

In this cohort study of 11 737 467 participants, the risk of GI cancer was significantly associated with drinking frequency. Compared with similar weekly alcohol consumption levels, the risk of GI cancer increased with a higher frequency of drinking but decreased with larger amounts per occasion.

**Meaning:**

This study suggests that frequent drinking may be a more important risk factor for incident GI cancers than the amount of alcohol consumed per occasion, and patients should be careful with their drinking habits, including regular consumption of small amounts of alcohol.

## Teaser

This cohort study uses data from the Korean National Health Insurance System database to evaluate the relative association of frequency of drinking vs amount of alcohol consumed per occasion with the development of gastrointestinal cancers.

## Introduction

Alcohol has been classified as a group 1 carcinogen by the International Agency of Research on Cancer.^1^ More than 5% of new cancer cases are associated with alcohol consumption.^2^ The gastrointestinal (GI) tract is one of the organs most easily affected by alcohol, and epidemiologic evidence shows links between alcohol consumption and the risk of cancers of the esophagus, colorectum, and liver^3^; it may also be associated with a higher risk of stomach and pancreatic cancer.^4,5^ The associations of alcohol consumption with biliary tract cancer are unclear.^6^

The link between drinking patterns and cancer risk has not been evaluated, even though drinking patterns vary significantly from person to person. Some people consume 1 to 2 units of beer, wine, or whisky every day (eg, frequent drinking), while other people enjoy several drinks at a bar or a party on the weekend only (eg, episodic binge drinking). However, most epidemiologic studies have focused only on total overall alcohol consumption, and data on the frequency and other dimensions of drinking patterns are lacking. For example, both 1 unit every day for 1 week and 7 units in 1 night produce a mean consumption of 1 drink a day for a 1-week period. One study reported that the increased frequency of drinking was associated with an increased risk of cancers in the colorectum, oral cavity, pharynx, larynx, liver, and esophagus in men but not in women, whereas heavy episodic drinking was associated with an increased risk of those cancers in women but not in men.^7^ However, that study focused on low- to moderate-volume drinkers. It was also limited by small outcome incidence (1611 cases of cancer for men and 1972 cases of cancer for women), limiting statistical power for individual cancers. Therefore, it is not clear which is more important: frequency of alcohol consumption or quantity of alcohol consumed per occasion.

In this context, we used a nationwide population-based cohort to investigate the association between drinking pattern and GI cancer development, over and above the association between total alcohol consumption and GI cancer development. In particular, we evaluated whether frequent drinking is more important than binge drinking in terms of incident overall and site-specific GI cancers.

## Methods

### Study Setting

The National Health Insurance is a major insurer in the Republic of Korea and covers approximately 97% of the Korean population, excluding only 3% of Medicaid beneficiaries. Information on use of medical facilities and a record of prescriptions with *International Statistical Classification of Diseases and Related Health Problems, Tenth Revision* (*ICD-10*) diagnosis codes under the National Health Insurance are gathered by the National Health Insurance Service (NHIS). In addition, the NHIS provides a free, biennial cardiovascular health screening for all beneficiaries 40 years or older and all employed individuals regardless of age, which consists of a self-administered questionnaire on health behavior (eg, past medical history, smoking, and drinking), anthropometric measurements (eg, body mass index [calculated as weight in kilograms divided by height in meters squared], and blood pressure), and a laboratory test (eg, fasting glucose and lipid levels).^8^ The NHIS also has information on demographic factors (eg, age, sex, place of residence, and income level) and links the data to a death registry database to manage qualification of the enrollees. The NHIS database has been used to establish cohort data for various epidemiologic studies.^9^ This cohort study was designed and conducted according to the Strengthening the Reporting of Observational Studies in Epidemiology (STROBE) reporting guideline.^10^ This study was approved by the institutional review board of Samsung Medical Center. The institutional review board waived the requirement for written informed consent from participants because the data are public and anonymized under confidentiality guidelines.

### Study Population

We initially included 12 724 396 individuals aged 40 years or older who underwent a health examination from January 1, 2009, to December 31, 2010. Individuals were excluded if (1) they had a history of any cancer before the health screening date (n = 303 424), (2) they had any missing information (n = 542 851), or (3) they had any cancer (n = 124 472) or died (n = 16 182) within 1 year after the health screening date. Finally, 11 737 467 eligible participants were followed up from 1 year after the health screening date to the date of any incident GI cancer, death, or the end of the study period (December 31, 2017), whichever came first.

### Exposure: Alcohol Intake Patterns

In the self-administered questionnaires, participants were asked to provide an approximate answer for frequency (number of days per week) and quantity (the amount of standard unit per occasion) of alcohol consumption. A standard unit was defined as a specialized cup for each type of alcohol such as beer, wine, Korean traditional alcohol (soju), or whisky.^11^ Although different drinks can have very different alcohol content, 1 standard unit contains roughly 8 g of ethanol in Korea.^11^ The exact methods to calculate the amount of alcohol consumed per each standard drink is described in the eMethods in the Supplement. Weekly alcohol consumption (g/week) was calculated as grams of alcohol per occasion multiplied by the frequency per week. Using the amount of alcohol intake per week, weekly consumption was classified into the following 4 categories: nondrinker (0 g/week), mild drinker (0-104 g/week), moderate drinker (105-209 g/week), and heavy drinker (≥210 g/week).^11^

### Outcome: Ascertainment of GI Cancer

The end point of the study was newly diagnosed GI cancer, defined as new claims with *ICD-10* C codes (for malignant neoplasm); these were also registered in the reliable rare incurable disease code for cancer (V193). Overall GI cancer was defined as *ICD-10* code C15-26, which included malignant neoplasms of the esophagus (code C15), stomach (code C16), colorectum (codes C18-20), liver (code C22), biliary tract (codes C23-24), and pancreas (code C25). The rare incurable disease system is a special copayment reduction program, implemented by the NHIS, to enhance health coverage and relieve the financial burden for patients with serious and rare diseases. For example, patients with cancer pay only 5% of the total medical bill incurred for cancer-related medical care. As enrollment in this program requires a medical certificate from a physician, the cancer diagnosis in our study is considered sufficiently reliable and has been used in previous studies.^12,13^

### Covariates

Income level was based on monthly insurance premium because insurance contributions are determined based on income level rather than health risk in Korea. Smoking status was classified into never smoker, ex-smoker (<20 pack-years or ≥20 pack-years), and current smoker (<20 pack-years or ≥20 pack-years) to account for smoking intensity. Regular exercise was defined as more than 30 minutes of moderate physical activity at least 5 times per week or more than 20 minutes of strenuous physical activity at least 3 times per week.^14^ Systolic and diastolic blood pressure were measured while the participant was in a seated position after at least 5 minutes of rest. Blood samples were obtained after overnight fasting. Comorbidities were based on claims data before the screening date and health examination results.

### Statistical Analysis

Statistical analysis was performed from January 1, 2019, to March 31, 2020. Baseline characteristics of participants according to weekly alcohol intake are presented as mean (SD) values for continuous variables and number and percentage for categorical variables. The independent *t* test for continuous variables and the χ^2^ test for categorical variables were used for statistical inference. Cox proportional hazards regression analysis was conducted to estimate hazard ratios (HRs) and 95% CIs for the association between alcohol consumption patterns and GI cancer development. Nondrinkers served as the reference group for all analyses. Model 1 was adjusted for age and sex. Model 2 was additionally adjusted for income level, smoking status with intensity, physical activity, body mass index, and the presence of type 2 diabetes. Model 3 was further adjusted for the presence of hypertension and dyslipidemia. For specific GI cancer incidence, we restricted analyses to each cancer site and performed the same analytic procedure. In addition, the relative association of the frequency and quantity of alcohol consumption within similar levels of alcohol consumption were assessed through a stratified analysis according to weekly alcohol consumption status. Statistical analyses were performed using SAS, version 9.4 (SAS Institute Inc), and a 2-sided *P* < .05 was considered statistically significant.

## Results

### Baseline Characteristics

The Table shows the baseline characteristics of the study population according to weekly alcohol consumption. At baseline, the prevalence of drinkers in the study population was 40.3%, which included mild drinkers (2 781 462 [23.7%]), moderate drinkers (n = 1 113 038 [9.5%]), and heavy drinkers (n = 832 635 [7.1%]). The mean (SD) alcohol intake per day was 6.2 (3.6) g for mild drinkers, 20.6 (3.9) g for moderate drinkers, and 51.5 (26.5) g for heavy drinkers. A comparison of heavy drinkers with nondrinkers revealed that heavy drinkers tended to be younger (mean [SD] age, 52.3 [9.5] vs 56.4 [11.0] years) and male (94.5% vs 28.9%), were current smokers (51.6% vs 9.0%), engaged in more regular exercise (moderate and heavy physical activity, 22.4% vs 18.2%), and had a higher mean (SD) body mass index (24.4 [3.0] vs 23.9 [3.1]). In addition, heavy drinkers had a higher prevalence of comorbid conditions compared with nondrinkers.

**Table.** Baseline Characteristics of Study Population According to Weekly Alcohol Consumption^a^

Variable	No. (%)			
	Nondrinkers (n = 7 010 332)	Drinkers		
		Mild (n = 2 781 462)	Moderate (n = 1 113 038)	Heavy (n = 832 635)
Age, mean (SD), y	56.4 (11.0)	51.8 (9.7)	51.5 (9.1)	52.3 (9.5)
Sex				
Men	2 027 999 (28.9)	1 805 061 (64.9)	992 950 (89.2)	786 681 (94.5)
Women	4 982 333 (71.1)	976 401 (35.1)	120 088 (10.8)	45 954 (5.5)
Income (lowest quartile)	1 883 641 (26.9)	671 687 (24.2)	243 933 (21.9)	186 379 (22.4)
Alcohol intake, mean (SD), g/d	NA	6.2 (3.6)	20.6 (3.9)	51.5 (26.5)
Smoking status				
Never	5 810 163 (82.9)	1 413 540 (50.8)	283 125 (25.4)	168 006 (20.2)
Ex-smoker				
<20 PY	330 639 (4.7)	426 874 (15.4)	190 356 (17.1)	116 989 (14.1)
≥20 PY	235 408 (3.4)	195 990 (7.1)	122 703 (11.0)	118 340 (14.2)
Current smoker				
<20 PY	281 892 (4.0)	404 652 (14.6)	227 469 (20.4)	128 900 (15.5)
≥20 PY	352 230 (5.0)	340 406 (12.2)	289 385 (26.0)	300 400 (36.1)
Physical activity				
None	5 736 859 (81.8)	2 165 900 (77.9)	859 791 (77.3)	646 113 (77.6)
Moderate	943 018 (13.5)	465 922 (16.8)	189 979 (17.1)	134 654 (16.2)
Heavy	330 455 (4.7)	149 640 (5.4)	63 268 (5.7)	51 868 (6.2)
BMI, mean (SD)	23.9 (3.1)	23.9 (2.9)	24.3 (2.9)	24.4 (3.0)
Waist circumference, mean (SD), cm	80.0 (8.7)	81.5 (8.4)	84.1 (7.7)	85.1 (7.8)
Blood pressure, mean (SD), mm Hg				
Systolic	123.4 (15.7)	123.5 (14.9)	126.8 (14.8)	128.3 (15.1)
Diastolic	76.1 (10.1)	77.2 (10.1)	79.6 (10.0)	80.5 (10.1)
Fasting glucose, mean (SD), mg/dL	98.9 (24.0)	99.4 (23.4)	103.0 (26.5)	105.8 (29.2)
Total cholesterol, mean (SD), mg/dL	199.8 (37.6)	198.1 (35.8)	199.0 (36.1)	198.4 (37.0)
Triglycerides, median (IQR), mg/dL	111.9 (111.8-111.9)	116.0 (115.9-116.1)	137.8 (137.6-137.9)	147.9 (147.7-148.1)
HDL-C, mean (SD), mg/dL	54.5 (17.3)	55.0 (15.4)	55.1 (15.5)	56.1 (16.6)
LDL-C, mean (SD), mg/dL	119.9 (34.3)	116.1 (33.1)	111.9 (34.2)	107.7 (35.3)
eGFR, mean (SD), mL/min/1.73 m^2^	84.6 (31.0)	86.7 (38.1)	88.3 (41.0)	89.7 (40.4)
Comorbidities				
Hypertension	2 406 414 (34.3)	829 585 (29.8)	404 736 (36.4)	334 289 (40.2)
Type 2 diabetes	832 222 (11.9)	274 386 (9.9)	141 392 (12.7)	126 937 (15.3)
Dyslipidemia	1 815 516 (25.9)	564 073 (20.3)	237 270 (21.3)	181 905 (21.9)
Chronic kidney disease	615 309 (8.8)	150 391 (5.4)	47 429 (4.3)	33 030 (4.0)
Ischemic heart disease	128 861 (1.8)	36 448 (1.3)	13 859 (1.3)	10 802 (1.3)
Stroke	413 277 (5.9)	85 355 (3.1)	29 409 (2.6)	23 340 (2.8)

Abbreviations: BMI, body mass index (calculated as weight in kilograms divided by height in meters squared); eGFR, estimated glomerular filtration rate; HDL-C, high-density lipoprotein cholesterol; IQR, interquartile range; LDL-C, low-density lipoprotein cholesterol; NA, not applicable; PY, pack-years.

SI conversion factors: To convert total cholesterol, HDL-C, and LDL-C to millimoles per liter, multiply by 0.0259; triglycerides to millimoles per liter, multiply by 0.0113; and glucose to millimoles per liter, multiply by 0.0555.

^a^ See the Methods section and eMethods in the Supplement for exact methods to calculate the amount of alcohol consumed per each standard drink.

### Alcohol Intake and GI Cancer

**Figure 1.**
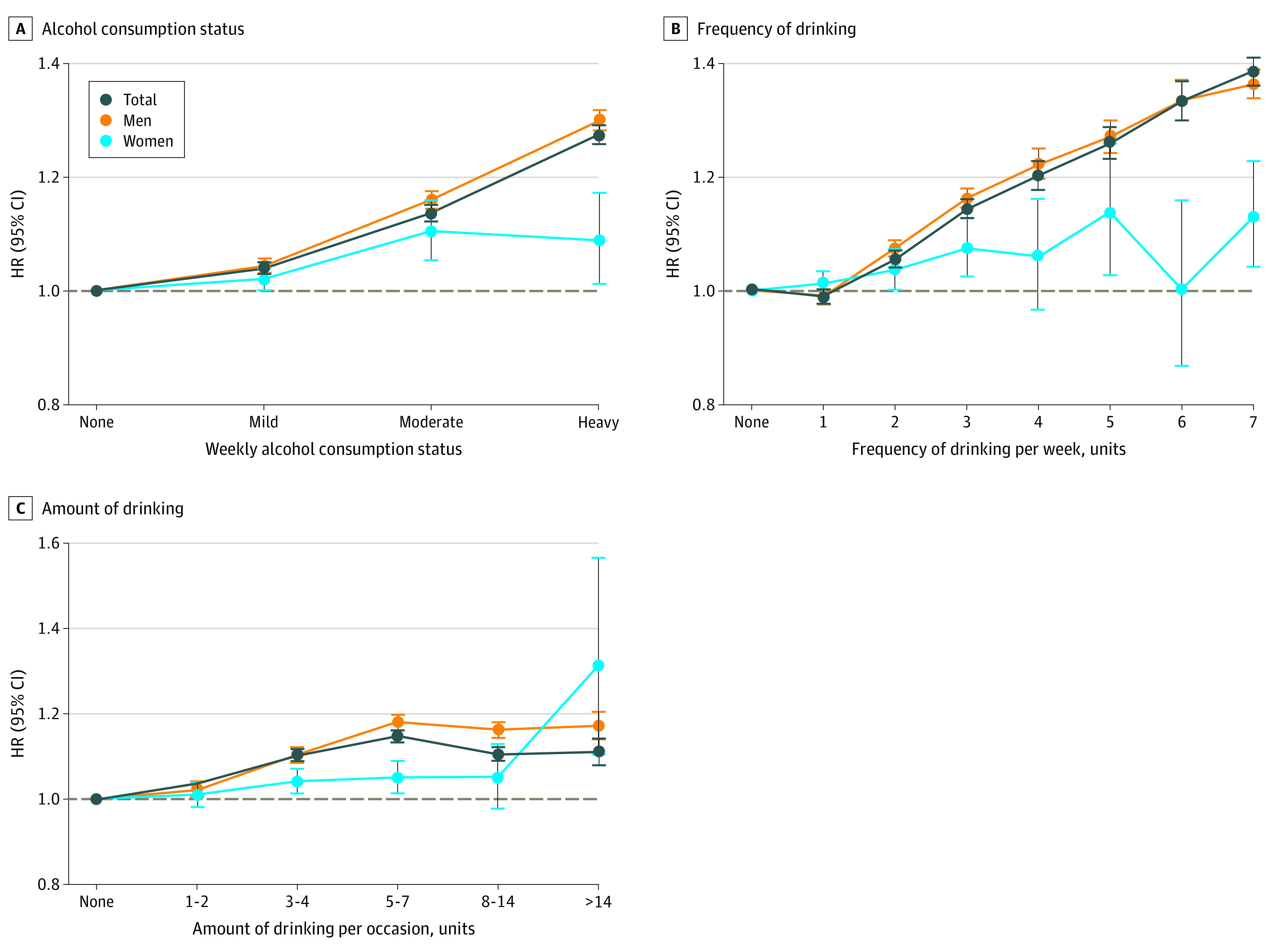
Risk of Gastrointestinal Cancer According to Alcohol Intake Pattern A, Weekly alcohol consumption status. B, Frequency of drinking per week. C, Amount (in units) of alcohol consumed per occasion. Hazard ratios (HRs) are adjusted for age, sex, income, smoking status with intensity, regular exercise, body mass index, diabetes, hypertension, and dyslipidemia. See the Methods section and eMethods in the Supplement for exact methods to calculate the amount of alcohol consumed per each standard drink. The horizontal dashed line in each panel indicates a reference line (HR, 1.0). Error bars indicate 95% CIs.

During a median of 6.4 years (interquartile range, 6.4-7.4 years) of follow-up, 319 202 participants (2.7%) developed GI cancer. Compared with nondrinkers, the risk of GI cancer was higher according to weekly consumption in mild drinkers (adjusted HR [aHR], 1.04; 95% CI, 1.03-1.05), moderate drinkers (aHR, 1.14; 95% CI, 1.12-1.15), and heavy drinkers (aHR, 1.28; 95% CI, 1.26-1.29) (Figure 1; eTables 1 and 2 in the Supplement). Risk patterns were generally similar for esophageal, gastric, colorectal, biliary, and pancreatic cancer; the exception was liver cancer, which showed a slightly decreased risk in mild drinkers (aHR, 0.91; 95% CI, 0.89-0.93) (eFigure and eTable 3 in the Supplement).

The risk of GI cancer increased linearly with the frequency of alcohol consumption in a dose-dependent manner (daily drinking: aHR, 1.39; 95% CI, 1.36-1.41) (Figure 1; eTable 1 in the Supplement). In contrast, the risk of GI cancer increased with consumption of up to 5 to 7 units per occasion (aHR, 1.15; 95% CI, 1.14-1.16), and participants with an alcohol intake per occasion greater than 5 to 7 units did not show increased HRs with increased intake per occasion (8-14 units per occasion: aHR, 1.11; 95% CI, 1.09-1.12; >14 units per occasion: aHR, 1.11; 95% CI, 1.08-1.14). Similar patterns were observed for combined strata of intake frequency and amount per occasion (eTables 4 and 5 in the Supplement) and for each specific GI cancer (eFigure and eTable 3 in the Supplement), including a linear increase with intake frequency and a weaker increase with number of units consumed per occasion.

Women showed a less prominent association than men between alcohol intake and the incidence of GI cancer for weekly consumption, frequency, and amount of alcohol consumed per occasion (Figure 1; eTables 6 and 7 in the Supplement). By cancer type, esophageal and liver cancers showed similar patterns between the sexes, while alcohol-related risk of colorectal, biliary, and pancreatic cancers was less prominent for women (eFigure in the Supplement).

### Alcohol Intake Pattern and GI Cancer: Frequency vs Amount per Occasion

**Figure 2.**
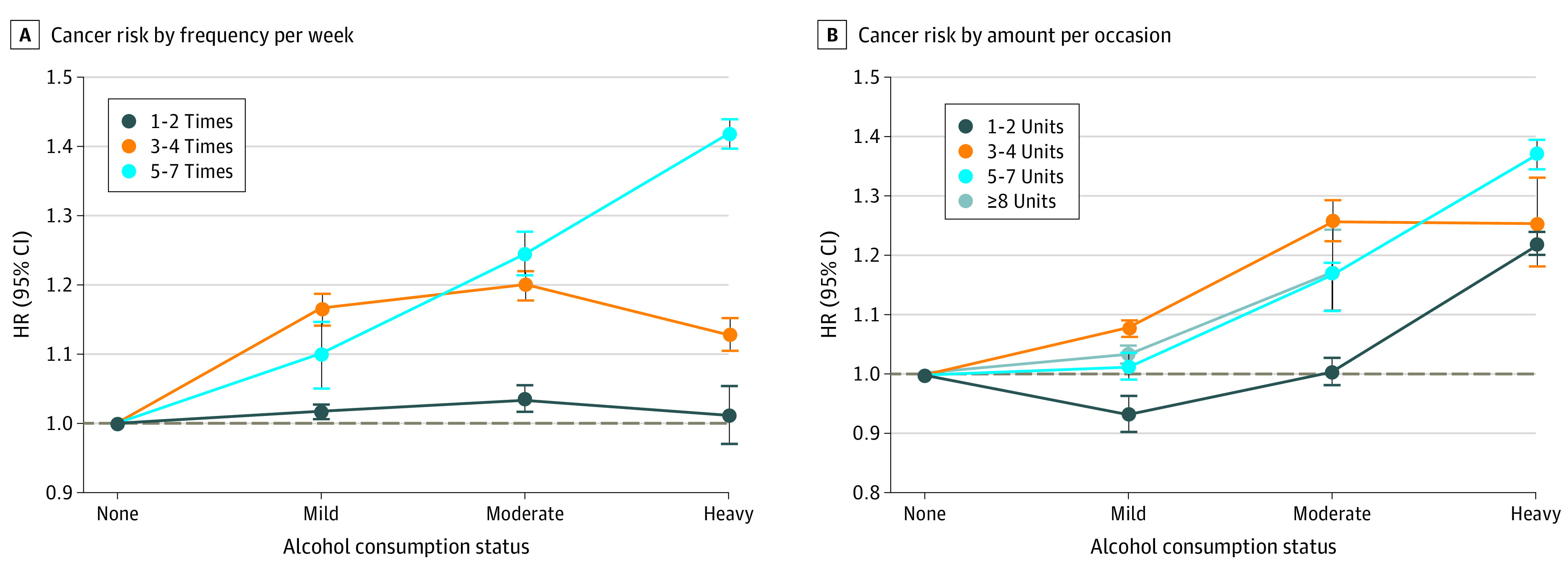
Association of Frequent Drinking vs Episodic Binge Drinking With Gastrointestinal Cancer Risk A, Cancer risk by frequency of drinking per week. B, Cancer risk by amount (in units) of alcohol consumed per occasion. Hazard ratios (HRs) are adjusted for age, sex, income, smoking status with intensity, regular exercise, body mass index, and diabetes. See the Methods section and eMethods in the Supplement for exact methods to calculate the amount of alcohol consumed per each standard drink. The horizontal dashed line in each panel indicates a reference line (HR, 1.0). Error bars indicate 95% CIs.

**Figure 3.**
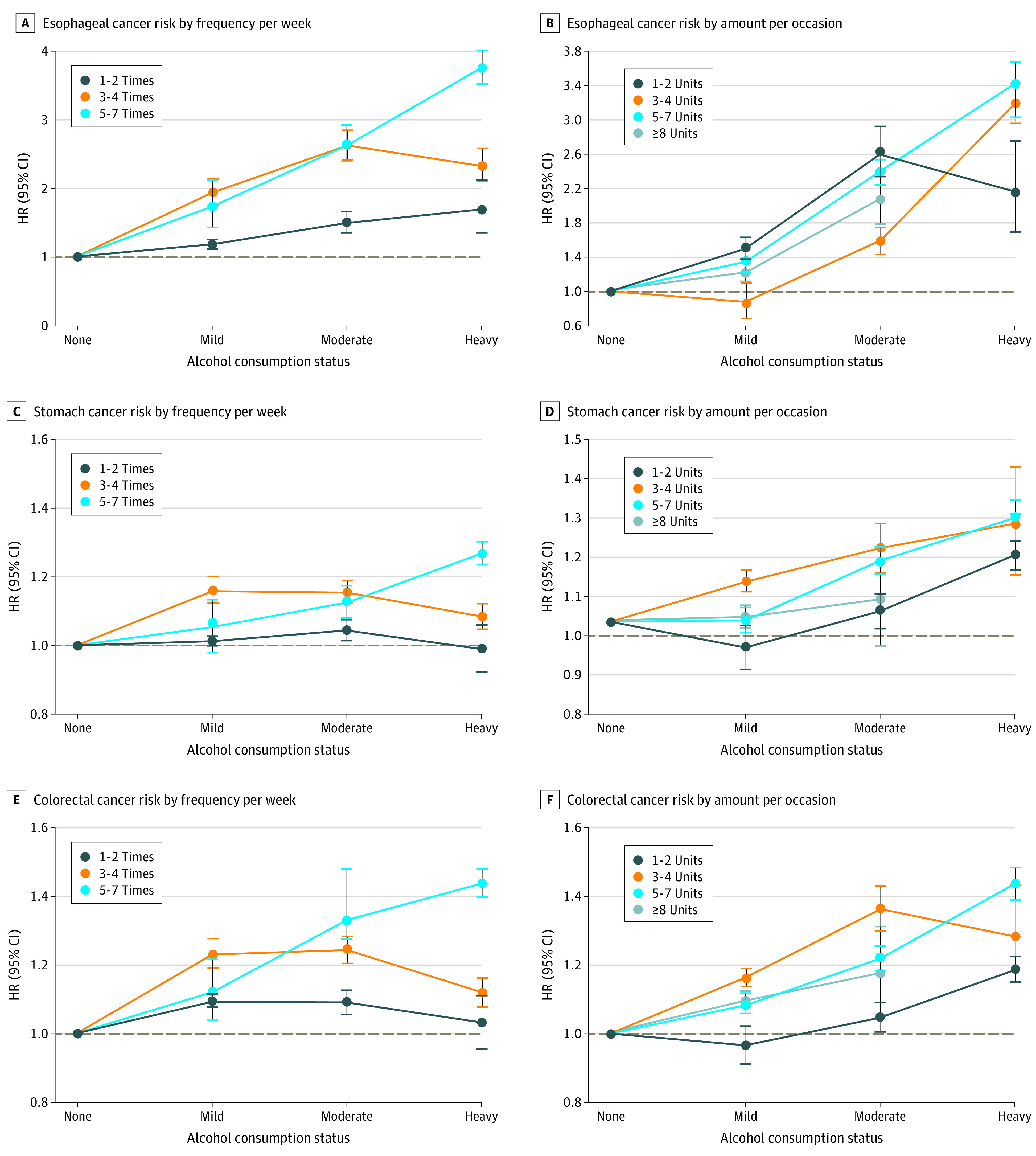
Association of Frequent Drinking vs Episodic Binge Drinking With Esophageal, Stomach, and Colorectal Cancer Risk A, Esophageal cancer risk by frequency of drinking per week. B, Esophageal cancer risk by amount (in units) of alcohol consumed per occasion. C, Stomach cancer risk by frequency of drinking per week. D, Stomach cancer risk by amount (in units) of alcohol consumed per occasion. E, Colorectal cancer risk by frequency of drinking per week. F, Colorectal cancer risk by amount (in units) of alcohol consumed per occasion. Hazard ratios (HRs) are adjusted for age, sex, income, smoking status with intensity, regular exercise, body mass index, and diabetes. See the Methods section and eMethods in the Supplement for exact methods to calculate the amount of alcohol consumed per each standard drink. The horizontal dashed line in each panel indicates a reference line (HR, 1.0). Error bars indicate 95% CIs.

**Figure 4.**
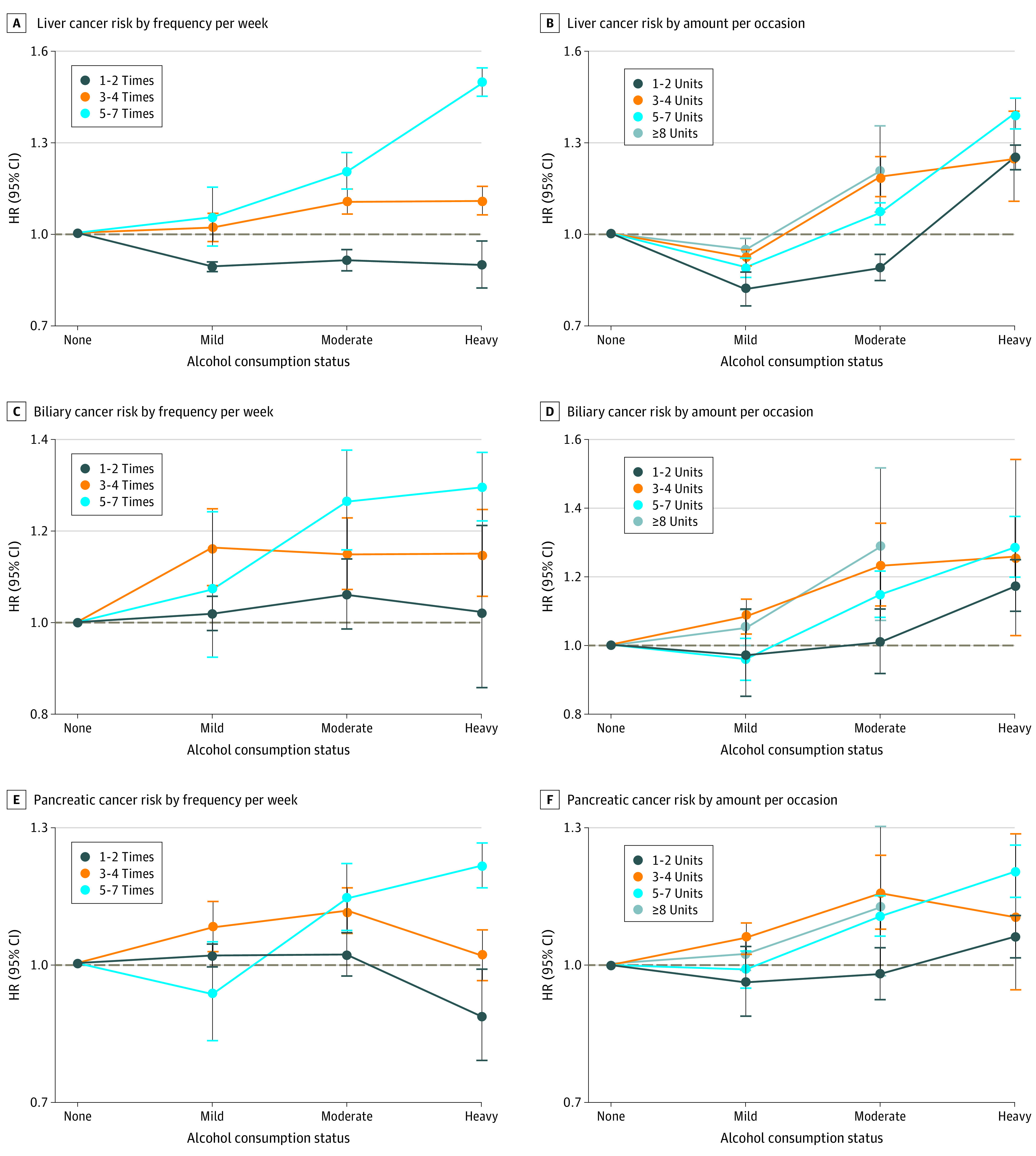
Association of Frequent Drinking vs Episodic Binge Drinking With Liver, Biliary, and Pancreatic Cancer Risk A, Liver cancer risk by frequency of drinking per week. B, Liver cancer risk by amount (in units) of alcohol consumed per occasion. C, Biliary cancer risk by frequency of drinking per week. D, Biliary cancer risk by amount (in units) of alcohol consumed per occasion. E, Pancreatic cancer risk by frequency of drinking per week. F, Pancreatic cancer risk by amount (in units) of alcohol consumed per occasion. Hazard ratios (HRs) are adjusted for age, sex, income, smoking status with intensity, regular exercise, body mass index, and diabetes. See the Methods section and eMethods in the Supplement for exact methods to calculate the amount of alcohol consumed per each standard drink. The horizontal dashed line in each panel indicates a reference line (HR, 1.0). Error bars indicate 95% CIs.

Figure 2 shows the results stratified by weekly alcohol consumption status (eTables 8 and 9 in the Supplement). When examined by frequency, GI cancer risk was highest in those who drank 3 to 4 times per week in the mild drinker group (aHR, 1.16; 95% CI, 1.14-1.18). In the moderate to heavy drinker group, the risk of GI cancer was higher with the frequency of alcohol consumption. When examined by amount per occasion, GI cancer risk was highest in those who drank 3 to 4 units per occasion in the mild drinker group (aHR, 1.07; 95% CI, 1.06-1.09), and the lowest risk was shown with the highest amount per occasion (>8 units) in each group (mild drinkers; aHR, 0.93; 95% CI, 0.90-0.96; moderate drinkers: aHR, 1.00; 95% CI, 0.98-1.02; and heavy drinkers: aHR, 1.21; 95% CI, 1.19-1.23). Specific cancer sites had a risk pattern similar to all GI cancers, showing a higher risk with higher frequency and a lower risk with higher amount per occasion within the strata, especially in moderate to heavy drinkers (Figure 3 and Figure 4; eTable 10 in the Supplement).

## Discussion

This study found an association between alcohol consumption and GI cancers, including stomach, pancreas, and biliary tract cancers, which was not established in previous studies, to our knowledge. We also showed that frequent drinking was more strongly associated than binge drinking with the risk of GI cancer and that this pattern was generally consistent across all GI cancer sites. Currently, a causal link has been established between alcohol consumption and cancers of the esophagus, colorectum, and liver,^2^ while association of other GI cancers with alcohol remains controversial. Alcohol consumption is considered a probable risk factor for stomach cancer based on evidence for intake greater than 45 g per day (approximately 3 units per day).^15^ The dose-response meta-analysis reported no significant association with stomach cancer risk per 10 g of ethanol per day, but it became statistically significant when 1 study with exceptionally high reported intakes of alcohol was excluded.^15^ Similarly, we showed a significant dose-response association between weekly alcohol consumption and stomach cancer risk. Thus, our study, performed in the area with the highest incidence of gastric cancer (39.6 per 100 000 persons vs 4.1 per 100 000 persons in the US),^16^ provides further supports for an association between alcohol consumption and gastric cancer.

Prior studies investigating the association between alcohol consumption and biliary tract cancer risk have been conflicting. Positive^17^ as well as null^18,19^ associations of alcohol consumption with biliary tract cancer have been reported. A recent meta-analysis reported null associations of alcohol consumption with biliary tract cancers, although it had high between-study heterogeneity.^6^ All studies included in the meta-analysis had insufficient statistical power owing to the small outcome incidences (gallbladder cancer, <218 cases; biliary cancer, <242 cases) as well as inability to control for important confounders.^6^ In our larger cohort study with high statistical power, results showed a dose-response association between weekly alcohol consumption and biliary cancer risk, which indicates a possible association between alcohol consumption and biliary tract cancer risk.

There is inconsistent evidence across the range of intakes for associations between alcohol consumption and pancreatic cancer. Consumption of more than about 3 units a day was associated with evidence of an increased risk of pancreatic cancers.^20^ Moreover, we showed that the risk of pancreatic cancer increased with weekly alcohol consumption, which also suggests a possible association between alcohol consumption and pancreatic cancer. In line with our study, a meta-analysis, which calculated the pooled relative risk limited to fully adjusted estimates only, consistently reported a significant 20% increase in the risk of pancreatic cancer with alcohol consumption.^4^

The novel finding of the current study is that frequent drinking may be more dangerous than binge drinking with regard to GI cancers. This finding suggests that repeated alcohol consumption events even at lower amounts of alcohol may have a greater carcinogenic effect on GI organs than the consumption of larger amounts of alcohol at a lower frequency. There is also very limited information on the mechanism by which frequency of drinking is an important determinant of cancer risk. However, it can be hypothesized based on the evidence to date as follows. First, regular alcohol consumption promotes the accumulation of cell divisions in the stem cells that maintain tissues in homeostasis.^21^ The division rates of stem cells probably increase above the usual rates (eg, every 21 days for the esophagus) when cytotoxic concentrations of ethanol are ingested regularly.^21^ When stem cells divide, they become exposed to unavoidable and carcinogen-mediated DNA alterations that increase their risk of malignant transformation.^21^ Second, repetitive and accumulated carcinogenic insult may result in carcinogenesis, whereas physiological homeostasis would be effective under conditions such as episodic alcohol exposure.^22^ Chronic exposure to acetaldehyde resulting from frequent drinking can lead to the formation of DNA adducts^23,24^ and induces chromosomal aberrations and sister chromatid exchanges in human lymphocytes.^10^ Acetaldehyde may also inhibit enzymes involved in DNA repair and ultimately lead to an impaired DNA damage response.^10,25^ Chronic alcohol consumption results in dose-dependent induction of cytochrome P450 2E1 (CYP2E1),^26^ which contributes to the hepatic oxidation of ethanol to acetaldehyde^10^; CYP2E1 also produces high quantities of reactive oxygen species that may cause DNA damage through oxidative stress, inflammation, and lipid peroxidation.^10,25^ Other pathologic characteristics associated with alcohol include tumor promotion activity, overproduction of mitogen-activated protein kinases, impaired expression of retinol and insulin-like growth factors, and immunosuppression.^10,25^ Third, the outcome of in vivo experiments supports that the association of alcohol with cancer development depends on duration of exposure as well as its dose.^25^ Regular alcohol exposure through frequent consumption might be associated with tumor initiation, while acute administration of alcohol, which mimics binge drinking, with enhanced tumor progression. Previous studies in mice reported that regular alcohol administration inhibited natural killer cells, which recognize and prevent neoplastic development. Although a single binge equivalent of alcohol also reduced natural killer cell number and lytic activity, prolonged alcohol exposure further activated monocytes and macrophages, resulting in chronic inflammation.^25^ More mechanistic research is required to unravel the intricate association between the metrics of alcohol exposure and cancer.

It has been suggested that an individual’s alcohol sensitivity and how they experience the effects of alcohol are associated with genotype. The aldehyde dehydrogenase (*ALDH2* [OMIM 100650]) gene encodes a major enzyme responsible for alcohol metabolism that eliminates acetaldehyde. The *ALDH2* polymorphism is associated with a flushing response to alcohol consumption and, consequently, generally is associated with lower alcohol consumption.^27^ The *ALDH2* polymorphisms also have a strong association with carcinogenic acetaldehyde accumulation after alcohol drinking.^28^ In this study, the habits of the participant subset who were mild-frequency or moderate-frequency drinkers but who were also binge drinkers, consuming more than 8 units of alcohol per occasion, might have a substantial overrepresentation of ALDH2*2 homozygotes and heterozygotes—they choose to drink alcohol but experience serious physiological consequences and often experience blackouts from drinking. Approximately 50% of East Asian individuals and 25% to 35% of Korean individuals have an inactive form of *ALDH2*, which is scarce in people of European descent.^27^ However, this study is based on data that were not originally designed for studying alcohol consumption; thus, we were not able to assess aldehyde dehydrogenase gene status among participants or identify major differences in the biology of Korean binge drinkers. Therefore, caution is required when applying our results to other ethnic groups.

Women differ from men in alcohol metabolism. Women have lower alcohol dehydrogenase activity in the stomach, which increases the bioavailability of alcohol.^29^ In addition, because women have less fluid in their bodies to distribute alcohol around, they have higher blood alcohol levels after drinking the same amount of alcohol as men.^29^ These factors result in a greater generation of toxic products, such as acetaldehyde and oxygen radicals, thereby contributing to the greater vulnerability of women to the effects of ethanol.^29^ We believe it is possible that women tend to avoid alcohol because many unpleasant reactions of alcohol are related to acetaldehyde accumulation in blood. In the present study, most moderate and heavy drinkers were men, and only 10.8% of moderate drinkers and 5.5% of heavy drinkers were women. Therefore, it is possible that the association of alcohol consumption with cancer risk in women was underestimated and appeared to be less prominent than in men.

In general, current guidelines on alcohol consumption specify only mean levels of consumption (eg, mean number of units consumed in 1 week). The American Cancer Society recommends that people who drink alcohol limit their intake to no more than 2 units per day for men and 1 drink a day for women,^30^ and the European Code Against Cancer recommends limiting or cutting out alcohol consumption.^31^ In addition to amount per occasion, our study provides a rationale for an emphasis on the frequency of drinking to prevent cancer. Alcohol users who have a glass of wine or beer during dinner every day may develop more cancer than people who occasionally consume several drinks.

### Strengths and Limitations

This study has some strengths. One major strength is that this is a cohort study using a population-based NHIS database, which is optimal for assessing the association between alcohol consumption pattern and GI cancers. Advantages of using such a population-based database include a large sample size (N = 11 737 467) that enabled us to secure enough statistical power for detailed analyses by sex, GI cancer site, and weekly alcohol intake; in addition, this nationwide population-based NHIS database allows near-complete follow-up.

Our study also has some limitations. First, we obtained lifestyle data, including alcohol consumption, based on self-administered questionnaires, and it is probable that people underreported their alcohol consumption.^32,33^ Second, because we used administrative data, we did not have sufficient clinical information, such as cancer histologic characteristics.

## Conclusions

In this large population-based cohort study, the frequency of drinking was a more important risk factor than the amount of alcohol consumed per occasion for incident GI cancers. These findings suggest that individuals should be counseled about regular low-dose alcohol use in addition to total amount of alcohol consumption or amount per occasion.

## Notes

Supplement.
eMethods.
**eTable 1.** Hazard Ratios and 95% Confidence Intervals for Gastrointestinal Cancer According to Drinking Status **eTable 2.** Hazard Ratios and 95% Confidence Intervals for Gastrointestinal (GI) Cancer According to Weekly Alcohol Consumption With 6 Groups **eTable 3.** Hazard Ratios and 95% Confidence Intervals by Site of Gastrointestinal (GI) Cancer According to Drinking Status **eTable 4.** Hazard Ratios and 95% Confidence Intervals for Gastrointestinal Cancer According to Combined Strata of Intake Frequency and Amount per Occasion **eTable 5.** The Effects of Frequency per Week and Amount per Occasion With Controlling for Each Other in Each Stratum by the Total Amount of Alcohol Consumption **eTable 6.** Hazard Ratios and 95% Confidence Intervals for Gastrointestinal (GI) Cancer According to Alcohol Intake Pattern–Frequent Drinking vs Binge Drinking (Men) **eTable 7.** Hazard Ratios and 95% Confidence Intervals for Gastrointestinal (GI) Cancer According to Alcohol Intake Pattern–Frequent Drinking vs Binge Drinking (Women) **eTable 8.** Hazard Ratios and 95% Confidence Intervals for Gastrointestinal Cancer According to Alcohol Intake Pattern, Frequent Drinking, or Episodic Binge Drinking **eTable 9.** Hazard Ratios and 95% Confidence Intervals for Gastrointestinal Cancer According to Alcohol Intake Pattern, Frequent Drinking, or Episodic Binge Drinking, Stratified by 6 Groups of Weekly Alcohol Consumption **eTable 10.** Hazard Ratios and 95% Confidence Intervals by Site of Gastrointestinal (GI) Cancer According to Alcohol Intake Pattern–Frequent Drinking vs Binge Drinking **eFigure.** The Risk of Site-Specific Gastrointestinal Cancer According to Alcohol Intake Pattern
