# Errors in Figures

**DOI:** 10.1001/jamanetworkopen.2021.30551

**Published:** 2021-09-23

In the Original Investigation titled “Association of the Frequency and Quantity of Alcohol Consumption With Gastrointestinal Cancer,”^1^ published August 18, 2021, the colors of some of the curves were incorrect in some of the panels in Figures 2, 3, and 4. The colors of these curves have been switched to correctly match their respective keys. This article has been corrected.^1^
