# Supplementary material for: Association of the Frequency and Quantity of Alcohol Consumption With Gastrointestinal Cancer
Source: JAMA Netw Open. 2021 Aug 18;4(8):e2120382. doi: 10.1001/jamanetworkopen.2021.20382 (PMC8374610; doi:10.1001/jamanetworkopen.2021.20382)
Supplement: Supplement. — eMethods. eTable 1. Hazard Ratios and 95% Confidence Intervals for Gastrointestinal Cancer According to Drinking Status eTable 2. Hazard Ratios and 95% Confidence Intervals for Gastrointestinal (GI) Cancer According to Weekly Alcohol Consumption With 6 Groups eTable 3. Hazard Ratios and 95% Confidence Intervals by Site of Gastrointestinal (GI) Cancer According to Drinking Status eTable 4. Hazard Ratios and 95% Confidence Intervals for Gastrointestinal Cancer According to Combined Strata of Intake Frequency and Amount per Occasion eTable 5. The Effects of Frequency per Week and Amount per Occasion With Controlling for Each Other in Each Stratum by the Total Amount of Alcohol Consumption eTable 6. Hazard Ratios and 95% Confidence Intervals for Gastrointestinal (GI) Cancer According to Alcohol Intake Pattern–Frequent Drinking vs Binge Drinking (Men) eTable 7. Hazard Ratios and 95% Confidence Intervals for Gastrointestinal (GI) Cancer According to Alcohol Intake Pattern–Frequent Drinking vs Binge Drinking (Women) eTable 8. Hazard Ratios and 95% Confidence Intervals for Gastrointestinal Cancer According to Alcohol Intake Pattern, Frequent Drinking, or Episodic Binge Drinking eTable 9. Hazard Ratios and 95% Confidence Intervals for Gastrointestinal Cancer According to Alcohol Intake Pattern, Frequent Drinking, or Episodic Binge Drinking, Stratified by 6 Groups of Weekly Alcohol Consumption eTable 10. Hazard Ratios and 95% Confidence Intervals by Site of Gastrointestinal (GI) Cancer According to Alcohol Intake Pattern–Frequent Drinking vs Binge Drinking eFigure. The Risk of Site-Specific Gastrointestinal Cancer According to Alcohol Intake Pattern [file jamanetwopen-e2120382-s001.pdf]

Supplementary Online Content

Yoo JE, Shin DW, Han K, et al. Association of the frequency and quantity of alcohol consumption with gastrointestinal cancer. *JAMA Netw Open.* 2021;4(8):e2120382. doi:10.1001/jamanetworkopen.2021.20382

eMethods.
eTable 1. Hazard Ratios and 95% Confidence Intervals for Gastrointestinal Cancer According to Drinking Status
eTable 2. Hazard Ratios and 95% Confidence Intervals for Gastrointestinal (GI) Cancer According to Weekly Alcohol Consumption With 6 Groups
eTable 3. Hazard Ratios and 95% Confidence Intervals by Site of Gastrointestinal (GI) Cancer According to Drinking Status
eTable 4. Hazard Ratios and 95% Confidence Intervals for Gastrointestinal Cancer According to Combined Strata of Intake Frequency and Amount per Occasion
eTable 5. The Effects of Frequency per Week and Amount per Occasion With Controlling for Each Other in Each Stratum by the Total Amount of Alcohol Consumption
eTable 6. Hazard Ratios and 95% Confidence Intervals for Gastrointestinal (GI) Cancer According to Alcohol Intake Pattern–Frequent Drinking vs Binge Drinking (Men)
eTable 7. Hazard Ratios and 95% Confidence Intervals for Gastrointestinal (GI) Cancer According to Alcohol Intake Pattern–Frequent Drinking vs Binge Drinking (Women)
eTable 8. Hazard Ratios and 95% Confidence Intervals for Gastrointestinal Cancer According to Alcohol Intake Pattern, Frequent Drinking, or Episodic Binge Drinking
eTable 9. Hazard Ratios and 95% Confidence Intervals for Gastrointestinal Cancer According to Alcohol Intake Pattern, Frequent Drinking, or Episodic Binge Drinking, Stratified by 6 Groups of Weekly Alcohol Consumption
eTable 10. Hazard Ratios and 95% Confidence Intervals by Site of Gastrointestinal (GI) Cancer According to Alcohol Intake Pattern–Frequent Drinking vs Binge Drinking
eFigure. The Risk of Site-Specific Gastrointestinal Cancer According to Alcohol Intake Pattern

This supplementary material has been provided by the authors to give readers additional information about their work.

© 2021 Yoo JE et al. *JAMA Network Open.*

eMethods.

1. In South Korea, one cup of beer contains 220 ml of beer. A cup used for Soju (Korean traditional alcohol) contains 50 ml of Soju.
2. We assume that a beer contains 4.5% alcohol and the specific gravity of alcohol is 0.79. For Soju, we assumed that it contains 21.0% alcohol.
3. One cup of beer contains 7.8 g of ethanol (220 * 0.045 * 0.79 = 7.821).
4. One cup of Soju contains 8.3 g of ethanol (50 * 0.21 * 0.79 = 8.295).
5. Finally, we assumed that 8 g of ethanol was consumed for one standard drink (one cup for each alcohol type).
6. Whisky has its own glass type that contains a similar amount of alcohol. However, the majority of alcohol consumed in South Korea is beer and Soju. In 2009, totals of 1,961,568 kiloliters of beer and 929,926 kiloliters of Soju were consumed. For whisky and wine, 4,351 and 22,633 kiloliters were consumed, respectively. Therefore, in contrast to Western countries, whisky and wine are minority beverages in South Korea; thus, we calculated alcohol consumption based on beer and Soju. These data can be found at the Korean Customs Service and Korean Alcohol Liquor Industry Association (http://www.kalia.or.kr/).
7. The questionnaire regarding alcohol consumption in 2009 Korean nationwide health screening is as follows (translated into English).

Please read the following questions and select your current situation.

On average, how many days a week do you drink?
☐ 0 ☐ 1 ☐ 2 ☐ 3 ☐ 4 ☐ 5 ☐ 6 ☐ 7

How much do you usually drink a day when you drink? (cups)

(※ We calculated amount according to the glass used for each liquor type, e.g., one can of beer (355cc) is equivalent to 1.6 cups of beer.)

© 2021 Yoo JE et al. *JAMA Network Open.*

eTable 1. Hazard Ratios and 95% Confidence Intervals for Gastrointestinal Cancer According to Drinking Status

Alcohol consumption	Subjects (N)	Events (n)	Person-years (PYs)	Incidence rate (per 1,000 PYs)	Model 1	Model 2	Model 3
**Weekly alcohol intake**							
Non-drinker	7,010,332	179,588	47,365,636.5	3.8	1 (ref.)	1 (ref.)	1 (ref.)
Mild drinker	2,781,462	71,782	18,881,467.5	3.8	**1.05 (1.04–1.06)**	**1.04 (1.03–1.05)**	**1.04 (1.03–1.05)**
Moderate drinker	1,113,038	35,202	7,520,125.4	4.7	**1.21(1.19–1.22)**	**1.14 (1.13–1.16)**	**1.14 (1.12–1.15)**
Heavy drinker	832,635	32,630	5,580,881.3	5.8	**1.39 (1.37–1.41)**	**1.29 (1.27–1.30)**	**1.28 (1.26–1.29)**
**Frequency (days per week)**							
0	7,010,332	179,588	47,365,636.5	3.8	1 (ref.)	1 (ref.)	1 (ref.)
1	1,897,327	42,324	12,929,920.4	3.3	1.00 (0.99–1.01)	0.99 (0.98–1.00)	**0.99 (0.98–1.00)**
2	1,224,552	32,983	8,312,396.5	4.0	**1.09 (1.08–1.11)**	**1.06 (1.05–1.07)**	**1.06 (1.04–1.07)**
3	787,753	25,574	5,314,067.0	4.8	**1.21 (1.19–1.23)**	**1.15 (1.14–1.17)**	**1.14(1.13–1.16)**
4	272,126	9,931	1,828,285.1	5.4	**1.29 (1.26–1.31)**	**1.21 (1.19–1.24)**	**1.20 (1.18–1.23)**
5	208,885	8,685	1,394,451.3	6.2	**1.36 (1.33–1.39)**	**1.27 (1.24,1.30)**	**1.26 (1.23–1.29)**
6	117,079	6,106	773,850.5	7.9	**1.44 (1.40–1.47)**	**1.35 (1.31–1.38)**	**1.34 (1.30–1.37)**
7	219,413	14,011	1,429,503.4	9.8	**1.48 (1.46–1.51)**	**1.40 (1.38–1.43)**	**1.39 (1.36–1.41)**
**Amount (units per occasion)**							
0	7,010,332	179,588	47,365,636.5	3.8	1 (ref.)	1 (ref.)	1 (ref.)
1–2	827,080	22,754	5,585,855.3	4.1	**1.03 (1.01–1.04)**	**1.04 (1.03–1.05)**	**1.04 (1.02–1.05)**
3–4	1,155,831	35,439	7,819,096.5	4.5	**1.13 (1.11–1.14)**	**1.11 (1.09–1.12)**	**1.10 (1.09–1.12)**
5–7	1,622,238	51,071	10,958,531.5	4.7	**1.22 (1.21–1.23)**	**1.15 (1.14–1.17)**	**1.15 (1.14–1.16)**
8–14	923,266	25,023	6,268,307.3	4.0	**1.19 (1.18–1.21)**	**1.11 (1.10–1.13)**	**1.11 (1.09–1.12)**
>14	198,720	5,327	1,350,683.6	3.9	**1.23 (1.19–1.26)**	**1.12 (1.09–1.15)**	**1.11 (1.08–1.14)**

Model 1: adjusted for age and sex
Model 2: adjusted for model 1 plus income, smoking status with intensity, regular exercise, body mass index, and diabetes mellitus
Model 3: adjusted for model 2 plus hypertension and dyslipidemia

© 2021 Yoo JE et al. *JAMA Network Open.*

eTable 2. Hazard Ratios and 95% Confidence Intervals for Gastrointestinal (GI) Cancer According to Weekly Alcohol Consumption With 6 Groups

Weekly alcohol consumption (g/week)		Subjects (N)	Events (n)	Person-years (PYs)	Incidence rate (per 1,000 PYs)	Model 1	Model 2	Model 3
Non-drinker		7,010,332	179,588	47,365,636.5	3.8	1 (ref.)	1 (ref.)	1 (ref.)
Mild drinker	<52.5	1,796,039	43,665	12,202,064.4	3.6	**1.02 (1.01–1.03)**	**1.02 (1.01–1.04)**	**1.02 (1.01–1.03)**
	52.5–105	985,423	28,117	6,679,403.1	4.2	**1.11 (1.10–1.13)**	**1.08 (1.06–1.09)**	**1.07 (1.06–1.09)**
Moderate drinker	105–210	1,113,038	35,202	7,520,125.4	4.7	**1.21 (1.20–1.23)**	**1.15 (1.13–1.16)**	**1.14 (1.13–1.16)**
Heavy drinker	210–315	393,986	13,442	2,653,212.0	5.1	**1.29 (1.26–1.31)**	**1.20 (1.18–1.22)**	**1.19 (1.17–1.21)**
	315–630	374,840	15,772	2,505,593.1	6.3	**1.44 (1.41–1.46)**	**1.32 (1.30–1.35)**	**1.31 (1.29–1.34)**
	≥630	63,809	3,416	422,076.2	8.1	**1.72 (1.66–1.78)**	**1.56 (1.51–1.61)**	**1.55 (1.49–1.60)**

Model 1: adjusted for age and sex
Model 2: adjusted for model 1 plus income, smoking status with intensity, regular exercise, body mass index, and diabetes mellitus
Model 3: adjusted for model 2 plus hypertension and dyslipidemia

© 2021 Yoo JE et al. *JAMA Network Open.*

eTable 3. Hazard Ratios and 95% Confidence Intervals by Site of Gastrointestinal (GI) Cancer According to Drinking Status

Alcohol intake pattern	Site of GI cancer					
	Esophagus	Stomach	Colorectal	Liver	Biliary	Pancreas
**Weekly alcohol intake**						
Non-drinker	1 (ref.)	1 (ref.)	1 (ref.)	1 (ref.)	1 (ref.)	1 (ref.)
Mild drinker	**1.34 (1.27–1.43)**	**1.04 (1.02–1.05)**	**1.12 (1.10–1.14)**	**0.91 (0.89–0.93)**	**1.04 (1.01–1.08)**	**1.03 (1.00–1.05)**
Moderate drinker	**2.23 (2.09–2.38)**	**1.11 (1.09–1.13)**	**1.20 (1.17–1.22)**	**1.03 (1.01–1.06)**	**1.14 (1.09–1.19)**	**1.08 (1.05–1.12)**
Heavy drinker	**3.20 (3.01–3.40)**	**1.18 (1.16–1.21)**	**1.29 (1.26–1.32)**	**1.29 (1.25–1.32)**	**1.23 (1.17–1.29)**	**1.12 (1.08–1.16)**
**Frequency (days per week)**						
0	1 (ref.)	1 (ref.)	1 (ref.)	1 (ref.)	1 (ref.)	1 (ref.)
1	**1.01 (0.93–1.09)**	0.99 (0.97–1.01)	**1.06 (1.04–1.08)**	**0.87 (0.85–0.89)**	1.00 (0.96–1.05)	1.00 (0.97–1.03)
2	**1.51 (1.41–1.63)**	**1.06 (1.04–1.08)**	**1.14 (1.11–1.17)**	**0.91 (0.88–0.93)**	**1.05 (1.00–1.10)**	**1.04 (1.01–1.07)**
3	**2.18 (2.03–2.34)**	**1.13 (1.10–1.15)**	**1.19 (1.16–1.22)**	**1.03 (1.00–1.06)**	**1.12 (1.06–1.18)**	**1.05 (1.02–1.09)**
4	**2.47 (2.25–2.72)**	**1.16 (1.13–1.21)**	**1.23 (1.19–1.28)**	**1.14 (1.09–1.19)**	**1.24 (1.15–1.34)**	**1.13 (1.07–1.19)**
5	**3.07 (2.81–3.37)**	**1.18 (1.14–1.22)**	**1.33 (1.29–1.38)**	**1.21 (1.16–1.27)**	**1.16 (1.06–1.26)**	**1.10 (1.04–1.17)**
6	**3.15 (2.85–3.49)**	**1.23 (1.17–1.28)**	**1.34 (1.28–1.41)**	**1.38 (1.31–1.45)**	**1.27 (1.15–1.39)**	**1.16 (1.09–1.25)**
7	**3.36 (3.12–3.60)**	**1.23 (1.19–1.27)**	**1.44 (1.40–1.49)**	**1.43 (1.38–1.48)**	**1.32 (1.24–1.41)**	**1.22 (1.17–1.28)**
**Amount (units per occasion)**						
0	1 (ref.)	1 (ref.)	1 (ref.)	1 (ref.)	1 (ref.)	1 (ref.)
1–2	**1.28 (1.17–1.39)**	**1.01 (0.99–1.04)**	**1.10 (1.08–1.13)**	**0.95 (0.92–0.98)**	**1.06 (1.01–1.11)**	1.03 (0.99–1.06)
3–4	**1.72 (1.61–1.84)**	**1.10 (1.08–1.12)**	**1.19 (1.17–1.22)**	**0.96 (0.93–0.98)**	**1.11 (1.06–1.16)**	**1.07 (1.04–1.10)**
5–7	**2.34 (2.21–2.48)**	**1.11 (1.09–1.13)**	**1.21 (1.19–1.23)**	**1.06 (1.04–1.09)**	**1.11 (1.06–1.16)**	**1.08 (1.05–1.11)**
8–14	**2.39 (2.22–2.58)**	**1.08 (1.06–1.11)**	**1.11 (1.09–1.14)**	**1.05 (1.02–1.08)**	**1.10 (1.03–1.16)**	1.02 (0.98–1.06)
>14	**2.77 (2.44–3.14)**	**1.07 (1.03–1.12)**	**1.07 (1.01–1.13)**	**1.15 (1.09–1.22)**	1.09 (0.97–1.22)	1.01 (0.94–1.09)

HRs are adjusted for age, sex, income, smoking status with intensity, regular exercise, body mass index, diabetes mellitus, hypertension, and dyslipidemia.

© 2021 Yoo JE et al. *JAMA Network Open.*

eTable 4. Hazard Ratios and 95% Confidence Intervals for Gastrointestinal Cancer According to Combined Strata of Intake Frequency and Amount per Occasion

Drinking frequency per week (days)	Amount consumed per occasion (units)	Subjects (N)	Events (n)	Person-years (PYs)	Incidence rate (per 1,000 PYs)	Model 1	Model 2	Model 3
0	0	7,010,332	179,588	47,365,636.5	3.8	1 (ref.)	1 (ref.)	1 (ref.)
1–2	1–2	688,315	16,682	4,670,423.8	3.6	1.00 (0.98–1.02)	1.01 (1.00–1.03)	1.01 (1.00–1.03)
	3–4	848,986	21,499	5,776,724.3	3.7	**1.05 (1.04–1.07)**	**1.04 (1.03–1.06)**	**1.04 (1.03–1.06)**
	5–7	975,191	24,207	6,630,481.5	3.7	**1.07 (1.06–1.09)**	**1.04 (1.02–1.05)**	**1.03 (1.02–1.05)**
	≥8	609,317	12,918	4,164,192.3	3.1	1.02 (1.00–1.04)	**0.96 (0.94–0.98)**	**0.96 (0.94–0.98)**
3–4	1–2	77,865	2,905	519,085.2	5.6	**1.11 (1.07–1.15)**	**1.11 (1.07–1.15)**	**1.11 (1.07–1.15)**
	3–4	207,587	8,438	1,390,532.5	6.1	**1.24 (1.21–1.26)**	**1.20 (1.17–1.23)**	**1.19 (1.17–1.22)**
	5–7	427,169	14,903	2,876,450.6	5.2	**1.29 (1.26–1.31)**	**1.20 (1.18–1.22)**	**1.20 (1.18–1.22)**
	≥8	347,245	9,259	2,356,190.5	3.9	**1.20 (1.17–1.22)**	**1.11 (1.08–1.13)**	**1.10 (1.08–1.12)**
5–7	1–2	60,829	3,166	395,844.0	8.0	**1.13 (1.09–1.17)**	**1.12 (1.08–1.16)**	**1.12 (1.08–1.16)**
	3–4	99,247	5,502	651,761.1	8.4	**1.30 (1.27–1.34)**	**1.25 (1.22–1.29)**	**1.25 (1.21–1.28)**
	5–7	219,875	11,961	1,451,577.8	8.2	**1.50 (1.47–1.53)**	**1.39 (1.37–1.42)**	**1.38 (1.36–1.41)**
	≥8	165,420	8,173	1,098,580.6	7.4	**1.61 (1.57–1.64)**	**1.46 (1.43–1.50)**	**1.45 (1.42–1.48)**

Model 1: adjusted for age and sex
Model 2: adjusted for model 1 plus income, smoking status with intensity, regular exercise, body mass index, and diabetes mellitus
Model 3: adjusted for model 2 plus hypertension and dyslipidemia

© 2021 Yoo JE et al. *JAMA Network Open.*

eTable 5. The Effects of Frequency per Week and Amount per Occasion With Controlling for Each Other in Each Stratum by the Total Amount of Alcohol Consumption

**Alcohol consumption**		**HR (95% CI)**
**Frequency (days per week)**		
Non-drinker	0	1 (ref.)
Mild drinker	1–2	**1.04 (1.03–1.06)**
	3–4	**1.16 (1.14–1.19)**
	5–7	**1.06 (1.02–1.11)**
Moderate drinker	1–2	**1.09 (1.06–1.11)**
	3–4	**1.23 (1.21–1.26)**
	5–7	**1.23 (1.19–1.26)**
Heavy drinker	1–2	**1.06 (1.01–1.12)**
	3–4	**1.18 (1.15–1.22)**
	5–7	**1.42 (1.39–1.45)**
**Amount (units per occasion)**		
Non–drinker	0	**1 (ref.)**
Mild drinker	1–2	**0.92 (0.90–0.94)**
	3–4	**0.97 (0.95–0.98)**
	5–7	**0.93 (0.92–0.95)**
	≥ 8	**0.88 (0.85–0.91)**
Moderate drinker	1–2	**0.78 (0.73–0.83)**
	3–4	**0.91 (0.87–0.94)**
	5–7	0.99 (0.97–1.01)
	≥ 8	**0.89 (0.87–0.91)**
Heavy drinker	3–4	**0.83 (0.78–0.89)**
	5–7	0.98 (0.94–1.01)
	≥ 8	**0.95 (0.92–0.97)**

HRs are adjusted for age, sex, income, smoking status with intensity, regular exercise, body mass index, diabetes mellitus, hypertension, dyslipidemia, and amount per occasion for frequency per week or frequency per week for amount per occasion.

© 2021 Yoo JE et al. *JAMA Network Open.*

eTable 6. Hazard Ratios and 95% Confidence Intervals for Gastrointestinal (GI) Cancer According to Alcohol Intake Pattern–Frequent Drinking vs Binge Drinking (Men)

Alcohol intake pattern		All GI cancer	Site of GI cancer					
			Esophagus	Stomach	Colorectal	Liver	Biliary	Pancreas
**Frequency (days per week)**								
Non-drinker	0	1 (ref.)	1 (ref.)	1 (ref.)	1 (ref.)	1 (ref.)	1 (ref.)	1 (ref.)
Mild drinker	1-2	**1.02 (1.01–1.03)**	**1.16 (1.08–1.24)**	**1.02 (1.00–1.04)**	**1.12 (1.10–1.15)**	**0.88 (0.86–0.91)**	**1.05 (1.01–1.10)**	**1.04 (1.01–1.07)**
	3-4	**1.16 (1.14–1.19)**	**1.91 (1.73–2.12)**	**1.17 (1.13–1.21)**	**1.23 (1.18–1.28)**	1.02 (0.97–1.07)	**1.20 (1.11–1.30)**	**1.10 (1.04–1.16)**
	5-7	**1.07 (1.02–1.12)**	**1.71 (1.40–2.10)**	1.02 (0.95–1.10)	1.08 (0.99–1.18)	1.05 (0.95–1.15)	1.05 (0.90–1.24)	0.91 (0.81–1.04)
Moderate drinker	1-2	**1.08 (1.06–1.10)**	**1.50 (1.35–1.66)**	**1.07 (1.04–1.10)**	**1.20 (1.16–1.24)**	**0.91 (0.87–0.94)**	**1.15 (1.07–1.24)**	**1.07 (1.02–1.13)**
	3-4	**1.22 (1.20–1.25)**	**2.61 (2.41–2.84)**	**1.17 (1.14–1.20)**	**1.30 (1.26–1.34)**	**1.10 (1.06–1.15)**	**1.21 (1.13–1.30)**	**1.15(1.09–1.20)**
	5-7	**1.22 (1.19–1.26)**	**2.60 (2.34–2.88)**	**1.11 (1.07–1.16)**	**1.31 (1.25–1.37)**	**1.20 (1.14–1.26)**	**1.23 (1.12–1.35)**	**1.12 (1.05–1.20)**
Heavy drinker	1-2	**1.06 (1.02–1.11)**	**1.72 (1.37–2.16)**	1.02 (0.95–1.09)	**1.14 (1.05–1.23)**	**0.90 (0.82–0.98)**	1.08 (0.90–1.29)	0.94 (0.84–1.05)
	3-4	**1.18 (1.15–1.21)**	**2.33 (2.10–2.58)**	**1.12 (1.08–1.16)**	**1.23 (1.18–1.28)**	**1.11 (1.07–1.16)**	**1.24 (1.14–1.35)**	**1.07 (1.01–1.13)**
	5-7	**1.42 (1.40–1.44)**	**3.72 (3.48–3.97)**	**1.27 (1.24–1.31)**	**1.45 (1.41–1.50)**	**1.49 (1.44–1.53)**	**1.33 (1.25–1.41)**	**1.22 (1.18–1.28)**
**Amount (units per occasion)**								
Non-drinker	0	1 (ref.)	1 (ref.)	1 (ref.)	1 (ref.)	1 (ref.)	1 (ref.)	1 (ref.)
Mild drinker	1-2	1.02 (1.00–1.04)	**1.20 (1.09–1.32)**	1.00 (0.98–1.03)	**1.06 (1.02–1.09)**	**0.95 (0.92–0.99)**	**1.08 (1.02–1.15)**	1.03 (0.99–1.08)
	3-4	**1.08 (1.06–1.10)**	**1.46 (1.35–1.58)**	**1.09 (1.07–1.12)**	**1.19 (1.16–1.22)**	**0.91 (0.88–0.94)**	**1.12 (1.06–1.18)**	**1.07 (1.03–1.11)**
	5-7	**1.04 (1.02–1.06)**	**1.33 (1.20–1.47)**	1.02 (1.00–1.05)	**1.17 (1.14–1.21)**	**0.89 (0.86–0.92)**	1.00 (0.93–1.07)	1.03 (0.98–1.07)
	≥ 8	0.98 (0.95–1.02)	0.88 (0.69–1.12)	0.98 (0.92–1.03)	**1.07 (1.01–1.14)**	**0.83 (0.77–0.89)**	1.05 (0.91–1.20)	1.01 (0.93–1.10)
Moderate drinker	1-2	**1.14 (1.07–1.21)**	**2.06 (1.61–2.62)**	**1.05 (0.94–1.16)**	1.12 (1.00–1.26)	**1.16 (1.02–1.31)**	1.22 (1.00–1.49)	1.09 (0.93–1.27)
	3-4	**1.24 (1.20–1.27)**	**2.56 (2.29–2.86)**	**1.15 (1.09–1.20)**	**1.34 (1.27–1.41)**	**1.19 (1.12–1.26)**	**1.21 (1.09–1.34)**	**1.13 (1.05–1.22)**
	5-7	**1.20 (1.18–1.22)**	**2.41 (2.23–2.61)**	**1.15 (1.12–1.18)**	**1.29 (1.25–1.33)**	**1.07 (1.03–1.11)**	**1.23 (1.15–1.30)**	**1.14 (1.10–1.19)**
	≥ 8	**1.06 (1.04–1.09)**	**1.59 (1.40–1.81)**	**1.06 (1.02–1.10)**	**1.19 (1.14–1.24)**	0.89 (0.85–0.94)	1.10 (1.00–1.21)	1.04 (0.98–1.10)
Heavy drinker	3-4	**1.23 (1.15–1.30)**	**2.14 (1.68–2.74)**	**1.19 (1.08–1.32)**	**1.25 (1.12–1.40)**	**1.25 (1.11–1.41)**	**1.27 (1.03–1.56)**	1.09 (0.93–1.27)
	5-7	**1.38 (1.35–1.40)**	**3.37 (3.13–3.64)**	**1.23 (1.19–1.27)**	**1.45 (1.40–1.50)**	**1.39 (1.34–1.44)**	**1.32 (1.23–1.42)**	**1.21 (1.16–1.27)**
	≥ 8	**1.27 (1.25–1.29)**	**3.19 (2.96–3.44)**	**1.18 (1.14–1.21)**	**1.30 (1.26–1.34)**	**1.26 (1.22–1.30)**	**1.25 (1.17–1.34)**	**1.11 (1.06–1.16)**

Hazard ratios are adjusted for age, income, smoking status with intensity, regular exercise, body mass index, and diabetes mellitus.

© 2021 Yoo JE et al. *JAMA Network Open.*

eTable 7. Hazard Ratios and 95% Confidence Intervals for Gastrointestinal (GI) Cancer According to Alcohol Intake Pattern–Frequent Drinking vs Binge Drinking (Women)

**Alcohol intake pattern**		**All GI cancer**	**Site of GI cancer**					
			**Esophagus**	**Stomach**	**Colorectal**	**Liver**	**Biliary**	**Pancreas**
**Frequency (days per week)**								
Non-drinker	0	1 (ref.)	1 (ref.)	1 (ref.)	1 (ref.)	1 (ref.)	1 (ref.)	1 (ref.)
Mild drinker	1-2	**1.02 (1.00–1.04)**	**1.47 (1.19 ,1.81)**	1.01 (0.97–1.04)	**1.06 (1.03–1.09)**	**0.93 (0.89–0.98)**	0.98 (0.92–1.04)	1.00 (0.95–1.04)
	3-4	1.05 (0.99–1.11)	**1.86 (1.11–3.12)**	1.02 (0.91–1.14)	1.07 (0.98–1.17)	1.02 (0.88–1.17)	0.90 (0.73–1.11)	0.94 (0.81–1.08)
	5-7	1.05 (0.93–1.19)	1.44 (0.46–4.48)	1.13 (0.92–1.41)	0.96 (0.79–1.18)	1.07 (0.81–1.42)	1.05 (0.73–1.51)	0.93 (0.69–1.24)
Moderate drinker	1-2	**1.04 (0.96–1.13)**	1.86 (0.92–3.79)	**1.15 (1.00–1.33)**	1.03 (0.92–1.17)	1.16 (0.96–1.39)	**0.68 (0.48–0.98)**	0.91(0.74–1.12)
	3-4	**1.14 (1.06–1.22)**	**2.49 (1.42–4.37)**	**1.16 (1.02–1.33)**	**1.19 (1.07–1.32)**	1.05 (0.88–1.26)	0.81 (0.60–1.10)	1.07 (0.89–1.27)
	5-7	**1.16 (1.05–1.27)**	**2.81 (1.45–5.45)**	1.06 (0.89–1.27)	1.04 (0.90–1.22)	1.21 (0.97–1.50)	**1.48 (1.13–1.93)**	1.20 (0.97–1.48)
Heavy drinker	1-2	**1.30 (1.02–1.67)**	-	1.51 (0.99–2.32)	**1.52 (1.08–2.15)**	0.94 (0.47–1.89)	1.82 (0.87–3.81)	0.91 (0.46–1.83)
	3-4	1.02 (0.90–1.16)	**2.54 (1.04–6.21)**	1.06 (0.84–1.34)	1.01 (0.83–1.22)	0.94 (0.68–1.30)	0.73 (0.42–1.25)	0.88 (0.63–1.21)
	5-7	**1.11 (1.01–1.23)**	**2.70 (1.38–5.28)**	1.05 (0.86–1.25)	1.00 (0.86–1.18)	**1.58 (1.30–1.92)**	0.81 (0.55–1.20)	1.10 (0.88–1.38)
**Amount (units per occasion)**								
Non-drinker	0	1 (ref.)	1 (ref.)	1 (ref.)	1 (ref.)	1 (ref.)	1 (ref.)	1 (ref.)
Mild drinker	1-2	1.01 (0.99–1.04)	1.22 (0.92–1.62)	0.99 (0.94–1.03)	**1.06 (1.03–1.11)**	**0.92 (0.87–0.98)**	0.97 (0.89–1.05)	0.97 (0.92–1.03)
	3-4	**1.04 (1.01–1.07)**	**1.85 (1.40–2.46)**	1.04 (0.99–1.10)	1.04 (1.00–1.09)	0.99 (0.92–1.07)	0.98 (0.88–1.08)	1.02 (0.95–1.09)
	5-7	1.02 (0.97–1.07)	**1.85 (1.18–2.89)**	1.00 (0.91–1.09)	1.04 (0.97–1.12)	0.90 (0.80–1.02)	0.99 (0.83–1.18)	0.96 (0.85–1.08)
	≥ 8	1.09 (0.96–1.24)	0.66 (0.09–4.74)	1.08 (0.85–1.37)	**1.24 (1.03–1.48)**	0.73 (0.50–1.07)	1.00 (0.61–1.64)	1.15 (0.85–1.55)
Moderate drinker	1-2	1.02 (0.84–1.22)	1.07 (0.15–7.61)	0.74 (0.50–1.10)	0.92 (0.68–1.26)	**1.65 (1.17–2.32)**	1.51 (0.95–2.40)	1.13 (0.76–1.69)
	3-4	**1.21 (1.09–1.35)**	**3.44 (1.71–6.94)**	1.13 (0.93–1.39)	1.11 (0.93–1.32)	1.12 (0.86–1.46)	**1.43 (1.04–1.97)**	1.27 (1.00–1.61)
	5-7	**1.11 (1.04–1.17)**	**2.22 (1.33–3.70)**	**1.17 (1.04–1.30)**	**1.15 (1.05–1.26)**	1.06 (0.91–1.23)	0.74 (0.56–0.96)	1.00 (0.86–1.16)
	≥ 8	1.06 (0.94–1.19)	**2.47 (1.01–6.02)**	1.20 (0.98–1.48)	0.98 (0.81–1.17)	1.15 (0.88–1.52)	0.81 (0.49–1.32)	0.93 (0.69–1.26)
Heavy drinker	3-4	0.95 (0.69–1.31)	-	0.94 (0.52–1.71)	**0.50 (0.25–0.99)**	0.84 (0.38–1.87)	0.77 (0.25–2.38)	0.93 (0.45–1.96)
	5-7	1.10 (0.99–1.23)	**2.91 (1.37–6.18)**	0.98 (0.79–1.23)	1.09 (0.91–1.29)	**1.58 (1.26–1.98)**	0.79 (0.50–1.24)	1.05 (0.80–1.37)
	≥ 8	1.11 (1.00–1.22)	**2.47 (1.16–5.28)**	1.18 (0.98–1.42)	1.07 (0.91–1.25)	1.13 (0.88–1.44)	0.93 (0.62–1.39)	0.98 (0.76–1.27)

Hazard ratios are adjusted for age, income, smoking status with intensity, regular exercise, body mass index, and diabetes mellitus.

© 2021 Yoo JE et al. *JAMA Network Open.*

eTable 8. Hazard Ratios and 95% Confidence Intervals for Gastrointestinal Cancer According to Alcohol Intake Pattern, Frequent Drinking, or Episodic Binge Drinking

**Alcohol intake pattern**		**Subjects (N)**	**Events (n)**	**Person-years (PYs)**	**Incidence rate (per 1,000 PYs)**	**Model 1**	**Model 2**	**Model 3**
**Frequency (days per week)**								
Non-drinker	0	7,010,332	179,588	47,365,636.5	3.8	1 (ref.)	1 (ref.)	1 (ref.)
Mild drinker	1–2	2,478,062	59,488	16,858,327.8	3.5	**1.03 (1.02-1.04)**	**1.02 (1.01-1.03)**	**1.02 (1.01–1.03)**
	3–4	262,156	10,260	1,754,012.7	5.8	**1.19 (1.16-1.21)**	**1.17 (1.14-1.19)**	**1.16 (1.14–1.18)**
	5–7	41,244	2,034	269,127.0	7.6	**1.10 (1.05-1.15)**	**1.10 (1.05-1.15)**	**1.09 (1.04–1.14)**
Moderate drinker	1–2	547,950	13,579	3,729,834.7	3.6	**1.09 (1.07-1.11)**	**1.04 (1.02-1.06)**	**1.03 (1.01–1.05)**
	3–4	438,236	15,022	2,955,851.6	5.1	**1.28 (1.25–1.30)**	**1.20 (1.18–1.22)**	**1.19 (1.17–1.21)**
	5–7	126,852	6,601	834,439.2	7.9	**1.29 (1.26–1.32)**	**1.25 (1.21–1.28)**	**1.24 (1.21–1.27)**
Heavy drinker	1–2	95,867	2,240	654,154.4	3.4	**1.09 (1.04–1.14)**	1.01 (0.97–1.05)	1.01 (0.97–1.05)
	3–4	359,487	10,223	2,432,488.0	4.2	**1.22 (1.20–1.25)**	**1.13 (1.11–1.15)**	**1.12 (1.10–1.15)**
	5–7	377,281	20,167	2,494,239.0	8.1	**1.54 (1.51–1.56)**	**1.42 (1.40–1.44)**	**1.41 (1.39–1.43)**
**Amount (units per occasion)**								
Non-drinker	0	7,010,332	179,588	47,365,636.5	3.8	**1 (ref.)**	**1 (ref.)**	
Mild drinker	1–2	807,489	21,622	5,459,096.5	4.0	**1.02 (1.01–1.04)**	**1.03 (1.02–1.05)**	**1.03 (1.02–1.05)**
	3–4	1,033,278	28,854	7,011,657.0	4.1	**1.09 (1.08–1.11)**	**1.08 (1.06–1.09)**	**1.07 (1.06–1.09)**
	5–7	740,175	17,366	5,037,642.0	3.4	**1.04 (1.03–1.06)**	**1.01 (1.00–1.03)**	1.01 (0.99–1.03)
	≥ 8	200,520	3,940	1,373,071.9	2.9	0.97 (0.94–1.00)	**0.93 (0.90–0.96)**	0.93 (0.90–0.96)
Moderate drinker	1–2	19,591	1,132	126,758.7	8.9	**1.19 (1.12–1.26)**	**1.17 (1.11–1.24)**	**1.17 (1.10–1.24)**
	3–4	104,558	5,478	690,010.8	7.9	**1.31 (1.27–1.34)**	**1.26 (1.22–1.29)**	**1.25 (1.22–1.29)**
	5–7	623,044	20,260	4,205,838.9	4.8	**1.25 (1.23–1.26)**	**1.17 (1.15–1.19)**	**1.16 (1.15–1.18)**
	≥ 8	365,845	8,332	2,497,517.0	3.3	**1.07 (1.04–1.09)**	1.00 (0.98–1.03)	1.00 (0.98–1.02)
Heavy drinker	3–4	17,995	1,107	117,428.8	9.4	**1.32 (1.24–1.40)**	**1.25 (1.18–1.33)**	**1.24 (1.17–1.32)**
	5–7	259,019	13,445	1,715,050.6	7.8	**1.48 (1.46–1.51)**	**1.37 (1.35–1.40)**	**1.36 (1.34–1.39)**
	≥ 8	555,621	18,078	3,748,402.0	4.8	**1.33 (1.31–1.35)**	**1.22 (1.20–1.24)**	**1.21 (1.19–1.23)**

Model 1: adjusted for age and sex
Model 2: adjusted for model 1 plus income, smoking status with intensity, regular exercise, body mass index, and diabetes mellitus
Model 3: adjusted for model 2 plus hypertension and dyslipidemia

© 2021 Yoo JE et al. *JAMA Network Open.*

eTable 9. Hazard Ratios and 95% Confidence Intervals for Gastrointestinal Cancer According to Alcohol Intake Pattern, Frequent Drinking, or Episodic Binge Drinking, Stratified by 6 Groups of Weekly Alcohol Consumption

**Alcohol intake pattern**			**Subjects (N)**	**Events (n)**	**Person-years (PYs)**	**Incidence rate (per 1,000 PYs)**	**Model 1**	**Model 2**	**Model 3**
**Frequency (days per week)**									
Non-drinker		0	7,010,332	179,596	47,365,642.4	3.8	1 (ref.)	1 (ref.)	1 (ref.)
Mild drinker (g/week)	<52.5	1–2	1,720,453	40,804	11,699,248.6	3.5	1.01 (1.00–1.02)	**1.01 (1.00–1.03)**	1.01 (1.00–1.02)
		3–4	65,962	2,408	439,941.9	5.5	**1.10 (1.06–1.15)**	**1.11 (1.06–1.15)**	**1.10 (1.06–1.15)**
		5–7	9,624	454	62,870.7	7.2	**1.13 (1.03–1.24)**	**1.13 (1.03–1.24)**	**1.13 (1.03–1.24)**
	52.5–105	1–2	757,609	18,686	5,159,075.9	3.6	**1.07 (1.05–1.08)**	**1.03 (1.01–1.05)**	**1.03 (1.01–1.04)**
		3–4	196,194	7,850	1,314,070.7	6.0	**1.21 (1.19–1.24)**	**1.18 (1.16–1.21)**	**1.18 (1.15–1.21)**
		5–7	31,620	1,578	206,256.3	7.7	**1.09 (1.04–1.15)**	**1.09 (1.03–1.14)**	**1.08 (1.03–1.14)**
Moderate drinker		1–2	547,950	13,581	3,729,834.3	3.6	**1.10 (1.08–1.12)**	**1.04 (1.02–1.06)**	**1.03 (1.02–1.05)**
		3–4	438,236	15,021	2,955,851.4	5.1	**1.28 (1.26–1.30)**	**1.20 (1.18–1.22)**	**1.19 (1.17–1.22)**
		5–7	126,852	6,601	834,438.9	7.9	**1.29 (1.26–1.32)**	**1.25 (1.22–1.28)**	**1.24 (1.21–1.27)**
Heavy drinker (g/week)	210–315	1–2	72,994	1,747	498,230.7	3.5	**1.10 (1.05–1.15)**	1.02 (0.98–1.07)	1.02 (0.97–1.07)
		3–4	208,521	6,093	1,409,236.2	4.3	**1.22 (1.19–1.25)**	**1.14 (1.11–1.17)**	**1.13 (1.10–1.16)**
		5–7	112,471	5,600	745,745.0	7.5	**1.43 (1.39–1.47)**	**1.33 (1.30–1.37)**	**1.32 (1.29–1.36)**
	315–630	1–2	22,156	472	151,072.1	3.1	1.06 (0.97–1.16)	0.98 (0.89–1.07)	0.97 (0.89–1.06)
		3–4	140,432	3,844	951,861.3	4.0	**1.23 (1.19–1.27)**	**1.13 (1.09–1.16)**	**1.12 (1.08–1.16)**
		5–7	212,252	11,454	1,402,658.4	8.2	**1.54 (1.51–1.57)**	**1.42 (1.39–1.45)**	**1.41 (1.38–1.44)**
	≥630	1–2	717	18	4,851.5	3.7	1.14 (0.73–1.79)	1.06 (0.67–1.66)	1.05 (0.67–1.65)
		3–4	10,534	284	71,390.2	4.0	**1.23 (1.10–1.38)**	1.11 (0.99–1.25)	1.10 (0.98–1.24)
		5–7	52,558	3,111	345,834.5	9.0	**1.78 (1.72–1.85)**	**1.62 (1.56–1.68)**	**1.60 (1.55–1.66)**
**Amount (units per occasion)**									
Non-drinker		0	7,010,332	179,596	47,365,642.4	3.8	1 (ref.)	1 (ref.)	1 (ref.)
Mild drinker (g/week)	<52.5	1–2	763,958	19,549	5,173,636.9	3.8	1.01 (1.00–1.03)	**1.03 (1.01–1.04)**	**1.03 (1.01–1.04)**
		3–4	741,743	18,217	5,049,053.4	3.6	**1.04 (1.02–1.06)**	**1.03 (1.02–1.05)**	**1.03 (1.02–1.05)**
		5–7	290,338	5,900	1,979,371.0	3.0	0.97 (0.94–0.99)	0.95 (0.93–0.98)	0.95 (0.92–0.97)
		1–2	43,531	2,074	285,457.3	7.3	**1.10 (1.05–1.15)**	**1.10 (1.05–1.14)**	**1.09 (1.04–1.14)**

© 2021 Yoo JE et al. *JAMA Network Open.*

	52.5–105	3–4	291,535	10,636	1,962,602.8	5.4	**1.19 (1.17–1.22)**	**1.16 (1.14–1.18)**	**1.15 (1.13–1.18)**
		5–7	449,837	11,464	3,058,270.9	3.7	**1.09 (1.06–1.11)**	**1.04 (1.02–1.06)**	**1.04 (1.02–1.06)**
		≥ 8	200,520	3,940	1,373,071.9	2.9	0.97 (0.94–1.00)	0.93 (0.90–0.96)	0.93 (0.90–0.96)
Moderate drinker		1–2	19,591	1,130	126,758.7	8.9	**1.19 (1.12–1.26)**	**1.17 (1.11–1.24)**	**1.16 (1.10–1.24)**
		3–4	104,558	5,478	690,010.5	7.9	**1.31 (1.27–1.35)**	**1.26 (1.23–1.30)**	**1.25 (1.22–1.29)**
		5–7	623,044	20,260	4,205,838.4	4.8	**1.24 (1.22–1.26)**	**1.17 (1.15–1.19)**	**1.16 (1.15–1.18)**
		≥ 8	365,845	8,333	2,497,517.0	3.3	**1.07 (1.04–1.09)**	1.00 (0.98–1.03)	1.00 (0.98–1.02)
Heavy drinker (g/week)	210–315	3–4	17,995	1,106	117,428.8	9.4	**1.31 (1.24–1.39)**	**1.25 (1.18–1.33)**	**1.24 (1.17–1.32)**
		5–7	159,629	7,051	1,065,137.1	6.6	**1.42 (1.39–1.46)**	**1.31 (1.28–1.35)**	**1.31 (1.27–1.34)**
		≥ 8	216,362	5,283	1,470,646.1	3.6	**1.13 (1.09–1.16)**	**1.05 (1.02–1.08)**	**1.04 (1.01–1.07)**
	315–630	5–7	99,390	6,391	649,913.4	9.8	**1.56 (1.52–1.60)**	**1.44 (1.41–1.48)**	**1.43 (1.40–1.47)**
		≥ 8	275,450	9,379	1,855,678.3	5.1	**1.36 (1.33–1.38)**	**1.24 (1.21–1.27)**	**1.23 (1.21–1.26)**
	≥630	5–7	63,809	3,413	422,076.2	8.1	**1.71 (1.65–1.77)**	**1.55 (1.50–1.60)**	**1.54 (1.49–1.59)**

Model 1: adjusted for age and sex
Model 2: adjusted for model 1 plus income, smoking status with intensity, regular exercise, body mass index, and diabetes mellitus
Model 3: adjusted for model 2 plus hypertension and dyslipidemia

© 2021 Yoo JE et al. *JAMA Network Open.*

eTable 10. Hazard Ratios and 95% Confidence Intervals by Site of Gastrointestinal (GI) Cancer According to Alcohol Intake Pattern–Frequent Drinking vs Binge Drinking

		**Site of GI cancer**					
		**Esophagus**	**Stomach**	**Colorectal**	**Liver**	**Biliary**	**Pancreas**
**Frequency (days per week)**							
Non-drinker	0	1 (ref.)	1 (ref.)	1 (ref.)	1 (ref.)	1 (ref.)	1 (ref.)
Mild drinker	1-2	**1.19 (1.12–1.27)**	1.01 (1.00–1.03)	**1.10 (1.08–1.12)**	**0.89 (0.87–0.91**)	1.02 (0.98–1.06)	1.02 (1.00–1.05)
	3-4	**1.94 (1.76–2.14)**	**1.16 (1.13–1.20)**	**1.23 (1.19–1.28)**	1.02 (0.98–1.07)	**1.16 (1.08–1.25)**	**1.08 (1.03–1.15)**
	5-7	**1.74 (1.43–2.13)**	1.06 (0.98–1.14)	**1.13 (1.04–1.21)**	1.05 (0.96–1.15)	1.07 (0.93–1.24)	0.94 (0.84–1.05)
Moderate drinker	1-2	**1.50 (1.35–1.67)**	**1.05 (1.02–1.08)**	**1.09 (1.06–1.13)**	**0.91 (0.88–0.95)**	1.06 (0.99–1.14)	1.02 (0.98–1.07)
	3-4	**2.63 (2.42–2.85)**	**1.16 (1.12–1.19)**	**1.25 (1.21–1.28)**	**1.11 (1.07–1.15)**	**1.15 (1.07–1.23)**	**1.12 (1.07–1.17)**
	5-7	**2.65 (2.39–2.93)**	**1.13 (1.08–1.18)**	**1.34 (1.28–1.40)**	**1.20 (1.14–1.27)**	**1.26 (1.16–1.38)**	**1.15 (1.08–1.22)**
Heavy drinker	1-2	**1.70 (1.35–2.13)**	0.99 (0.92–1.06)	1.03 (0.96–1.11)	**0.90 (0.82–0.98)**	1.02 (0.86–1.21)	**0.89 (0.79–0.99)**
	3-4	**2.33 (2.11–2.58)**	**1.09 (1.05–1.12)**	**1.12 (1.08–1.16)**	**1.11 (1.06–1.16)**	**1.15 (1.06–1.25)**	1.02 (0.97–1.08)
	5-7	**3.76 (3.53–4.01)**	**1.27 (1.24–1.30)**	**1.44 (1.40–1.48)**	**1.50 (1.45–1.54)**	**1.29 (1.22–1.37)**	**1.22 (1.17–1.27)**
**Amount (units per occasion)**							
Non-drinker	0	1 (ref.)	1 (ref.)	1 (ref.)	1 (ref.)	1 (ref.)	1 (ref.)
Mild drinker	1-2	**1.23 (1.12–1.34)**	1.01 (0.99–1.04)	1.10 (1.07–1.13)	**0.95 (0.92–0.98)**	1.05 (1.00–1.10)	1.02 (0.99–1.06)
	3-4	**1.50 (1.39–1.62)**	**1.09 (1.07–1.11)**	**1.16 (1.14–1.19)**	**0.92 (0.90–0.95)**	**1.08 (1.03–1.13)**	**1.06 (1.02–1.09)**
	5-7	**1.35 (1.22–1.49)**	1.00 (0.98–1.03)	**1.09 (1.06–1.12)**	**0.89 (0.86–0.92)**	0.96 (0.90–1.02)	0.99 (0.95–1.03)
	≥ 8	0.87 (0.68–1.10)	**0.94 (0.90–0.99)**	0.97 (0.91–1.02)	**0.82 (0.77–0.88)**	0.97 (0.85–1.11)	0.96 (0.89–1.04)
Moderate drinker	1-2	**2.08 (1.63–2.64)**	1.05 (0.95–1.16)	**1.18 (1.06–1.31)**	**1.21 (1.07–1.36)**	**1.29 (1.07–1.55)**	1.13 (0.98–1.30)
	3-4	**2.62 (2.34–2.92)**	**1.16 (1.11–1.22)**	**1.37 (1.30–1.43)**	**1.19 (1.12–1.26)**	**1.23 (1.12–1.36)**	**1.16 (1.08–1.24)**
	5-7	**2.42 (2.24–2.61)**	**1.13 (1.11–1.16)**	**1.22 (1.19–1.26)**	**1.07 (1.04–1.10)**	**1.15 (1.08–1.22)**	**1.11 (1.06–1.15)**
	≥ 8	**1.59 (1.40–1.81)**	1.02 (0.99–1.06)	**1.05 (1.01–1.09)**	**0.89 (0.85–0.93)**	1.01 (0.92–1.11)	0.98 (0.92–1.04)
Heavy drinker	3-4	**2.16 (1.69–2.76)**	**1.22 (1.10–1.34)**	**1.28 (1.15–1.43)**	**1.25 (1.11–1.40)**	**1.26 (1.03–1.54)**	1.10 (0.95–1.29)
	5-7	**3.41 (3.16–3.68)**	**1.23 (1.19–1.27)**	**1.44 (1.39–1.49)**	**1.39 (1.35–1.45)**	**1.28 (1.20–1.38)**	**1.20 (1.15–1.26)**
	≥ 8	**3.19 (2.96–3.43)**	**1.15 (1.12–1.18)**	**1.19 (1.15–1.22)**	**1.25 (1.21–1.29)**	**1.17 (1.10–1.25)**	**1.06 (1.02–1.11)**

Hazard ratios are adjusted for age, income, smoking status with intensity, regular exercise, body mass index, and diabetes mellitus.

© 2021 Yoo JE et al. *JAMA Network Open.*

eFigure. The Risk of Site-Specific Gastrointestinal Cancer According to Alcohol Intake Pattern

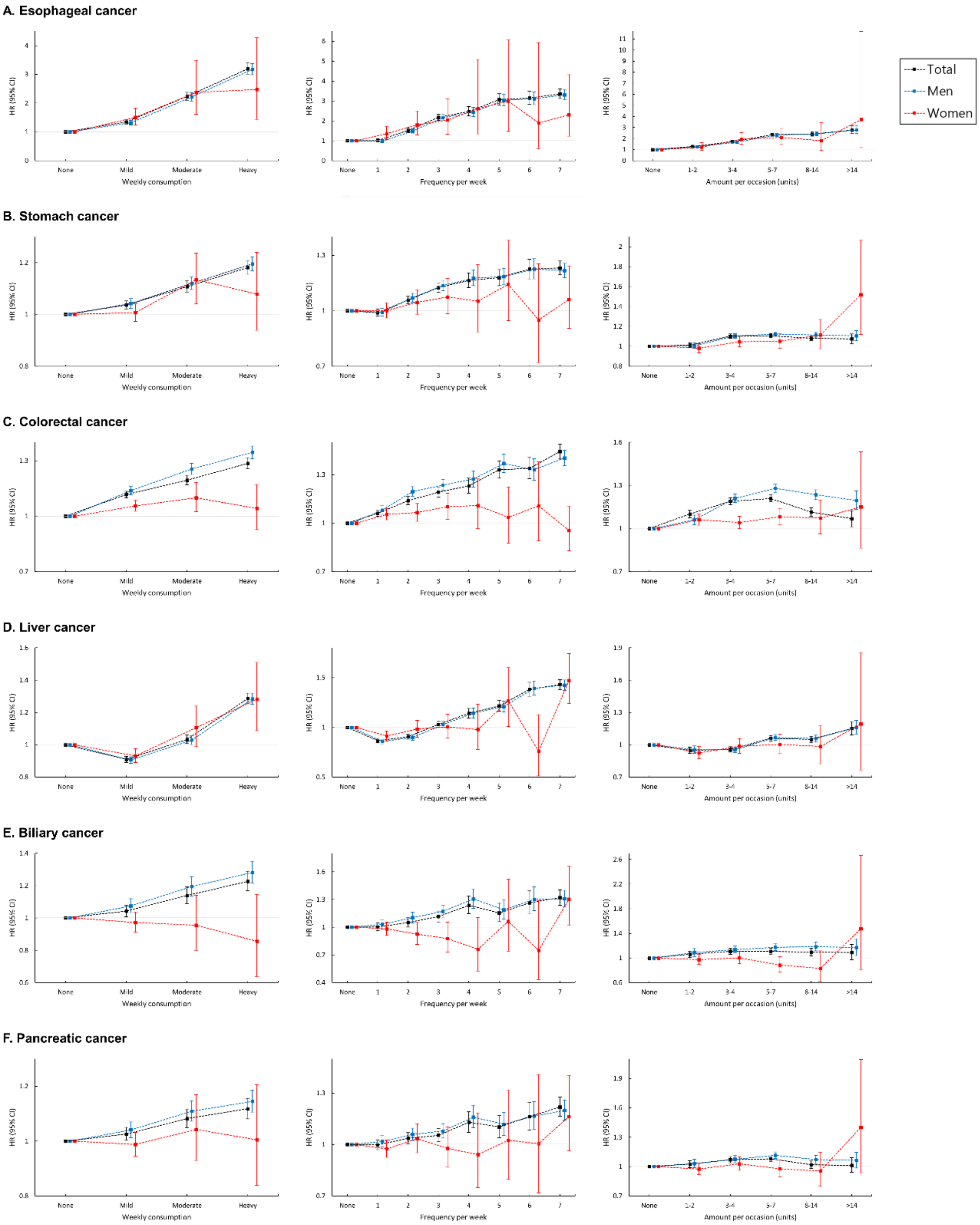

HR, hazard ratio; CI, confidence interval

HRs are adjusted for age, sex, income, smoking status with intensity, regular exercise, body mass index, diabetes mellitus, hypertension, and dyslipidemia.

© 2021 Yoo JE et al. *JAMA Network Open*.
